# Pancreatic Cancer: Pathogenesis and Clinical Studies

**DOI:** 10.1002/mco2.70162

**Published:** 2025-04-02

**Authors:** Kexun Zhou, Yingping Liu, Chuanyun Tang, Hong Zhu

**Affiliations:** ^1^ Department of Medical Oncology Cancer Center West China Hospital Sichuan University Chengdu China; ^2^ Department of Radiotherapy Cancer Hospital Chinese Academy of Medical Sciences Beijing China; ^3^ The First Clinical Medical College of Nanchang University Nanchang University Nanchang China; ^4^ Division of Abdominal Tumor Multimodality Treatment Cancer Center West China Hospital Sichuan University Chengdu China

**Keywords:** pancreatic ductal adenocarcinoma, gene alteration, signaling pathways, treatment development, personalized management

## Abstract

Pancreatic cancer (PC) is a highly lethal malignancy, with pancreatic ductal adenocarcinoma (PDAC) being the most common and aggressive subtype, characterized by late diagnosis, aggressive progression, and resistance to conventional therapies. Despite advances in understanding its pathogenesis, including the identification of common genetic mutations (e.g., KRAS, TP53, CDKN2A, SMAD4) and dysregulated signaling pathways (e.g., KRAS–MAPK, PI3K–AKT, and TGF‐β pathways), effective therapeutic strategies remain limited. Current treatment modalities including chemotherapy, targeted therapy, immunotherapy, radiotherapy, and emerging therapies such as antibody–drug conjugates (ADCs), chimeric antigen receptor T (CAR‐T) cells, oncolytic viruses (OVs), cancer vaccines, and bispecific antibodies (BsAbs), face significant challenges. This review comprehensively summarizes these treatment approaches, emphasizing their mechanisms, limitations, and potential solutions, to overcome these bottlenecks. By integrating recent advancements and outlining critical challenges, this review aims to provide insights into future directions and guide the development of more effective treatment strategies for PC, with a specific focus on PDAC. Our work underscores the urgency of addressing the unmet needs in PDAC therapy and highlights promising areas for innovation in this field.

## Graphical abstract

Although the treatment landscape for cancer has evolved rapidly in recent years, progress has stagnated for pancreatic cancer (PC). PC includes multiple types, but pancreatic ductal adenocarcinoma (PDAC) accounts for >90% of cases. This review focuses on the molecular pathogenesis and different treatments for PDAC, hoping to propose future directions for optimizing treatment modalities and improving clinical outcomes of PDAC (created with BioRender.com).

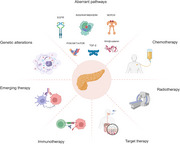

## 1 Introduction

Pancreatic cancer (PC) ranks among the most lethal malignancies globally. It is a heterogeneous disease encompassing multiple subtypes, the most prevalent of which is pancreatic ductal adenocarcinoma (PDAC), accounting for nearly 90% of cases. Other types include pancreatic neuroendocrine tumor, and intraductal papillary mucinous neoplasm [1]. PDAC, the focus of this review, is notorious for its asymptomatic early stage, complex molecular landscape, and resistance to conventional therapies. Over the past decades, significant advancements in molecular biology and imaging technologies have profoundly expanded the understanding of PDAC, especial unveiling its complex interplay between genetic predispositions and environmental risk factors. Nevertheless, PDAC remains a leading cause of cancer‐related mortality, with a 5‐year survival rate approximately 10%, highlighting the need for more effective therapies [2].

Recent research has increasingly focused on elucidating the complex mechanisms underlying pancreatic tumorigenesis, encompassing key genetic alterations (e.g., KRAS, TP53 mutations), the dynamic role of the tumor microenvironment (TME), and the strategies employed by tumors to evade immune surveillance [3, 4, 5]. Clinically, the advent of novel therapeutic approaches, including targeted therapies and immunotherapies, has demonstrated potential. However, formidable challenges persist. This review aims to provide a comprehensive overview of the pathogenesis of PDAC, with an emphasis on the molecular and genetic drivers. Also, we explore the latest developments in various therapies, evaluating their potential to transform clinical outcomes. Furthermore, the review will highlight the challenges and limitations in translating basic science into clinical practice, emphasizing the need for personalized treatment.

The structure of this review is as follows: the first section will explore the molecular pathogenesis of PC, with a particular emphasis on pivotal genetic alterations and dysregulated signaling pathways that drive tumorigenesis. Subsequently, we will synthesize recent advancements in therapeutic strategies, focusing on systemic treatment modalities and clinical challenges. Finally, we will discuss emerging therapies, including novel targeted agents and immunotherapeutic approaches, while delineating future research directions to address the unmet needs in managing this highly aggressive malignancy. Collectively, this review aims to provide a comprehensive and nuanced perspective on the current landscape of PDAC, while identifying critical knowledge gaps that warrant further investigation to advance therapeutic outcomes.

## 2 Pathogenesis of PDAC

Understanding the molecular mechanisms of PDAC is crucial for improving early diagnosis, developing targeted therapies, and formulating personalized treatment.

### 2.1 Genetic Alterations

The development of PDAC is closely associated with genetic mutations, which drive cancer initiation, progression, and metastasis. Most PDACs exhibit specific patterns of genetic mutations, with common alterations including KRAS, TP53, CDKN2A, and SMAD4. These mutations not only affect the proliferative, migratory, and antiapoptotic capabilities of cancer cells but may also influence their response to treatment.

KRAS mutations are the most frequent in PDAC, occurring in approximately 90% of cases [6]. KRAS, a small GTPase, is involved in various cellular processes such as proliferation, differentiation, and migration. Mutations in KRAS typically lead to its constitutive activation, promoting abnormal progression. Common mutation sites include codons 12, 13, and 61, with codon 12 (e.g., p.G12D, p.G12V) being particularly prevalent [3]. These mutations impair the intrinsic GTPase activity of KRAS, maintaining it in an active state and continuously activating downstream signaling pathways, including MAPK and PI3K/AKT [7]. Due to the widespread presence of KRAS mutations, the development of targeted therapies, such as KRAS G12C inhibitors, has become a focus. However, the unique structure of the KRAS protein presents significant challenges in developing therapeutic agents.

TP53 mutations represent the second most prevalent genetic alteration in PDAC, occurring in approximately 70% of cases [8]. The TP53 gene encodes the p53 protein, a pivotal tumor suppressor that orchestrates critical cellular processes, including cell cycle regulation, DNA repair, and apoptosis induction. In PDAC, TP53 mutations predominantly manifest as point mutations, particularly within the DNA‐binding domain, leading to the functional inactivation of p53 and subsequent loss of its tumor‐suppressive capabilities [9, 10]. While p53 is a key tumor suppressor, restoring its function is complex, and therapeutic strategies aiming to reactivating p53 remain a significant challenge. Recent studies have explored small molecules and gene editing techniques to restore the activity of p53, but clinical applications are still in the early stages [11].

CDKN2A, located on chromosome 9, is a critical tumor suppressor gene that regulates the G1/S phase transition via p16INK4a protein [12, 13]. Mutations or deletions of CDKN2A are common in PDAC, present in about 60% of cases [8]. These alterations lead to the loss of p16INK4a function, relieving its inhibition on CDK4/6 and promoting progression into the S phase, thereby facilitating tumor cell proliferation. CDKN2A mutations can serve as diagnostic markers for PDAC, particularly in familial cases, where their detection aids in the early identification of high‐risk individuals [14, 15].

SMAD4 is a transcription factor involved in the TGF‐β signaling pathway. Its mutation occurs in approximately 40% of PDAC cases [8]. These mutations impair SMAD4's ability to regulate the TGF‐β pathway, affecting cellular behaviors such as proliferation, migration, and resistance to apoptosis [16]. Although no direct targeted therapies for SMAD4‐deficient PDAC currently exist, modulating the TGF‐β pathway with inhibitors may provide a novel therapeutic approach.

Despite the identification of several mutations closely associated with PDAC development, therapies against these mutations face substantial challenges. The intrinsic structural complexity of the KRAS protein has hindered the development of direct inhibitors. Although the recent emergence of KRAS G12C inhibitors represents a significant breakthrough, their clinical efficacy is constrained by intrinsic and acquired resistance mechanisms. Moreover, PDAC is a highly heterogeneous disease, which makes it difficult for a single targeted therapy to achieve comprehensive efficacy. Thus, personalized treatment remain a significant challenge.

### 2.2 Aberrant Signaling Pathways

**FIGURE 1:**
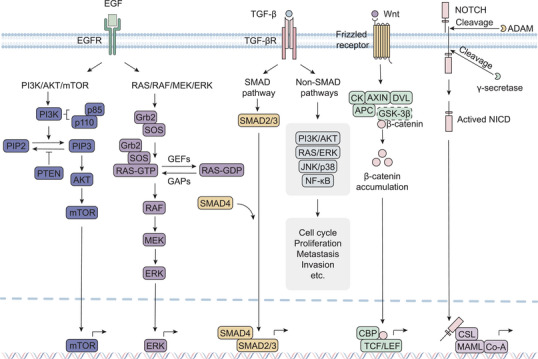
Important pathways and their functions in pancreatic ductal adenocarcinoma. EGFR is expressed on the surface of normal epithelial cells and is frequently overexpressed in pancreatic cancer cells, contributing to tumor progression. EGFR signaling primarily activates two major downstream pathways: the RAS/RAF/MEK/ERK pathway, which regulates cell proliferation and survival, and the PI3K/AKT/mTOR pathway, which controls cell growth and metabolism. The TGF‐β pathway mediates its effects through both canonical (SMAD‐dependent) and noncanonical (SMAD‐independent) mechanisms. Upon TGF‐β binding to its receptor TGFβR, receptor‐associated kinases phosphorylate and activate SMAD2/3. Activated SMAD2/3 then forms a complex with SMAD4, which translocates to the nucleus to regulate the transcription of target genes. Additionally, TGF‐β signaling can activate non‐SMAD pathways, including PI3K/AKT, RAS/ERK, JNK/p38, and NF‐κB, which collectively modulate diverse cellular processes. In the Wnt signaling pathway, the binding of Wnt ligands to Frizzled receptors activates DVL. Activated DVL induces the dissociation of GSK‐3β from the AXIN complex, thereby inhibiting GSK‐3β activity. This inhibition prevents β‐catenin phosphorylation, leading to its cytoplasmic accumulation. Subsequently, stabilized β‐catenin translocates to the nucleus, where it binds to TCF/LEF transcription factors to activate the transcription of Wnt target genes involved in cell proliferation and differentiation. The NOTCH signaling pathway is initiated when NOTCH ligands bind to NOTCH receptors, triggering sequential proteolytic cleavage mediated by ADAM and γ‐Secretase. This process releases the NICD, which translocates to the nucleus and binds to the CSL transcription factor. NICD recruits MAML proteins, displacing transcriptional corepressors and recruiting Co‐A to promote the transcription of NOTCH target genes. *Abbreviations*: DVL, disheveled; GSK‐3β, glycogen synthase kinase‐3β; CBP, Creb‐binding protein; TCF, beta‐catenin‐T‐cell factor; LEF, lymphoid enhancer factor; NICD, NOTCH intracellular domain; CSL, CBF1/Su(H)/Lag‐1; MAML, mastermind‐like; Co‐A, coactivators (Created with Adobe illustrator).

The development of PDAC involves the dysregulation of multiple signaling pathways. Commonly disrupted pathways in PDAC include EGFR, RAS/RAF/MEK/ERK, TGF‐β, Wnt/β‐catenin, PI3K/AKT/mTOR, and NOTCH (Figure 1).

#### 2.2.1 EGFR Pathway

EGFR is a transmembrane receptor belonging to the receptor tyrosine kinase (RTK) family. Upon ligand binding, EGFR undergoes dimerization and autophosphorylation of its intracellular tyrosine residues, which activates multiple downstream signaling cascades that regulate critical cellular processes [17]. Additionally, EGFR signaling upregulates the expression of various proteins involved in metastasis, such as matrix metalloproteinases (MMPs) and E‐cadherin. MMPs degrade the extracellular matrix (ECM), thereby facilitating tumor cell migration and invasion, while E‐cadherin modulation promotes epithelial–mesenchymal transition (EMT), further enhancing the migratory and invasive potential of cancer cells [18, 19]. These synergistic mechanisms collectively contribute to the aggressive biological behavior of PDAC.

The activation of EGFR also plays a pivotal role in drug resistance. Corcoran et al. [20] investigated the sensitivity of BRAF V600E‐mutant cancer cell lines to RAF inhibition. The results suggested that, rapid EGFR‐mediated reactivation of the MAPK pathway significantly contributes to resistance to RAF inhibitor (vemurafenib) [20]. In addition, the activation of EGFR pathway is associated with the upregulation of PI3K/AKT/p65 pathway, which, in turn, reduces chemosensitivity [21]. Furthermore, EGFR‐mediated resistance is not limited to intrinsic activation of alternative pathways, the TME also plays a critical role. Hypoxic conditions, aberrant angiogenesis, and dynamic stromal interactions can significantly modulate EGFR signaling, thereby contributing to treatment insensitivity and therapeutic failure. This multifaceted interplay highlights the complexity of EGFR resistance mechanisms and underscores the need for comprehensive therapeutic strategies targeting both tumor cells and their microenvironment [22, 23, 24].

#### 2.2.2 RAS/RAF/MEK/ERK Pathway

The RAS family genes, including KRAS, NRAS, and HRAS, are frequently mutated proto‐oncogenes in various cancers, particularly in PDAC. These mutations drive tumor initiation and progression by activating downstream signaling pathways, with the RAF/MEK/ERK pathway being one of the most crucial [25, 26]. Upon activation, RAS binds and activates RAF kinase, which in turn phosphorylate and activate MEK1/2. This leads to the activation of ERK1/2, whose nuclear translocation regulates the expression of genes involved in cell proliferation and differentiation [27]. In PDAC, continuous MAPK pathway activation promotes aggressive tumor characteristics [16].

In addition to its effects on tumor cells, persistent MAPK activation profoundly impacts the TME. The ERK pathway contributes to ECM remodeling, facilitating tumor cell invasion and metastasis [28]. Moreover, MAPK signaling fosters immune evasion. For example, dendritic cells (DCs) dysfunction is associated with the activation of MAPK, which contributes to the suppression of T‐cell response [29]. And its activation regulates the expression of PD‐L1, promoting immunosuppression [30]. Additionally, MAPK activation induces key transcription factors, such as snail and twist, which drive EMT and enhance the invasive potential of tumor cells, further exacerbating aggressiveness [31, 32].

Given its pivotal role in PDAC, the RAS/RAF/MEK/ERK pathway is an attractive target for therapeutic intervention. However, its complexity and the heterogeneity of PDAC pose significant challenges. Tumors often exhibit genetic diversity, and MAPK signaling can be activated through multiple mechanisms, complicating the design of targeted therapies and increasing the risk of resistance. For instance, tumors that initially respond to targeted inhibitors may acquire resistance through secondary mutations or compensatory activation of alternative pathways. To address these challenges, current research is increasingly focused on the development of combination strategies that simultaneously target multiple nodes within the MAPK signaling cascade, often in synergy with conventional chemotherapy or immunotherapy. Moreover, the design of highly selective inhibitors targeting specific RAF isoforms or MEK/ERK kinases holds significant promise, as these agents are anticipated to exhibit enhanced efficacy while minimizing off‐target effects. These advancements collectively represent a paradigm shift in treatment strategies, offering renewed optimism for achieving better clinical outcomes.

#### 2.2.3 PI3K/AKT/mTOR Pathway

The PI3K/AKT/mTOR pathway also significantly contributes to the progression of PDAC. This pathway acts as a central regulator of tumor cell proliferation, primarily through the inhibition of key cell cycle regulators, including p21 and p27, which function as critical suppressors of uncontrolled cell division. By downregulating these tumor suppressors, the PI3K/AKT/mTOR axis drives aberrant cell cycle progression and facilitates unchecked proliferation [33]. Moreover, beyond its role in proliferation, this pathway plays a pivotal role in metabolic reprogramming, enabling tumor cells to adapt their metabolism to meet the heightened demands for energy and biosynthetic precursors. Specifically, mTOR activation orchestrates essential anabolic processes by promoting protein synthesis, modulating lipid metabolism, and enhancing glucose uptake, thereby supporting the rapid growth of cancer cells [34].

PI3K/AKT/mTOR signaling also contributes to chemoresistance via inhibiting apoptotic pathways. AKT activation phosphorylates and inactivates proapoptotic proteins like p53 and Bad, preventing apoptosis even in the presence of chemotherapy [35]. Also, PI3K/AKT/mTOR can regulate the expression of PD‐L1 on tumor cells, suppressing T cell‐mediated cytotoxicity [36]. Additionally, PI3K/AKT/mTOR signaling alters the immune landscape by promoting the recruitment of immunosuppressive cells, such as regulatory T cells (Tregs), which further dampen antitumor immunity and facilitate tumor progression [37].

Although targeting the PI3K/AKT/mTOR pathway in PDAC has demonstrated promising results in preclinical models, its translational potential is significantly constrained by the pathway's inherent complexity and robust compensatory mechanisms. The functional heterogeneity of PI3K isoforms, AKT isoforms, and mTOR complexes, coupled with genetic alterations both upstream and downstream of the pathway, often results in the limited efficacy of single‐target inhibitors.

#### 2.2.4 TGF‐β Pathway

TGF‐β is a pivotal cytokine that orchestrates downstream signaling via its transmembrane receptors and the SMAD protein family. In the early stages of PDAC, TGF‐β signaling functions as a potent tumor suppressor. Upon ligand binding, TGF‐β activates SMAD2 and SMAD3, which form a complex with SMAD4 and translocate to the nucleus. This complex subsequently modulates the transcription of target genes involved in cell cycle arrest and apoptosis, thereby inhibiting uncontrolled cellular proliferation and constraining tumor progression. However, as the disease advances, TGF‐β signaling undergoes a functional switch to foster tumorigenic processes. This phenotypic transition is predominantly mediated by the dysregulation of key molecular mechanisms, such as the induction of EMT [38].

In PDAC, dysregulation of the TGF‐β signaling is commonly due to mutations, deletions, or aberrant expression of downstream molecules. SMAD4, a critical transcription factor in the TGF‐β pathway, typically transduces tumor‐suppressive signals. In approximately 40% of PDAC, mutations of the SMAD4 gene impair this tumor‐suppressive function, thereby promoting tumor progression [8]. Additionally, aberrant expression of TGF‐β receptors, such as the overexpression of TGF‐βR I and II, disrupts normal signaling, reducing the tumor‐suppressive effects of TGF‐β and amplifying its protumorigenic impact [39, 40].

Given its pivotal role in PDAC pathogenesis, TGF‐β has emerged as a promising therapeutic target. However, the dualistic nature of TGF‐β poses significant challenges in the development of effective agents. Excessive inhibition of TGF‐β signaling may lead to unintended adverse effects (AEs), particularly in tissues where TGF‐β regulates immune homeostasis and tissue repair. For instance, suppression of TGF‐β signaling has been associated with the induction of autoimmune responses, highlighting the need for precise intervention [41].

#### 2.2.5 Wnt/β‐Catenin Pathway

The Wnt/β‐catenin signaling is a pivotal regulator of diverse biological processes. Under physiological conditions, its activity is tightly regulated by tumor suppressors, which prevent the cytoplasmic accumulation of β‐catenin. However, somatic mutations in adenomatous polyposis coli, Axin, or other components of the Wnt signaling cascade lead to the persistent stabilization and nuclear translocation of β‐catenin. This aberrant activation drives the transcriptional upregulation of oncogenes, including c‐Myc and cyclin D1, which are critical mediators of tumor cell proliferation [42, 43, 44].

Beyond its role in promoting tumor proliferation, Wnt/β‐catenin signaling plays a central role in maintaining cancer stem cells (CSCs), a subpopulation characterized by self‐renewal capacity and pluripotency. CSCs are key drivers of tumor initiation, recurrence, and metastasis, and their inherent resistance to conventional therapies represents a major challenge in cancer treatment. Wnt/β‐catenin signaling sustains the CSC pool by upregulating stem cell markers, such as leucine‐rich repeat‐containing G‐protein coupled receptor 5 (Lgr5), thereby enhancing tumor invasiveness and metastatic potential [45, 46].

Targeting the Wnt/β‐catenin signaling is complicated, due to its complex and multifaceted regulation. In addition to somatic mutations in adenomatous polyposis coli and Axin, the pathway is modulated by extracellular inhibitors, including Dickkopf proteins and secreted Frizzled‐related proteins (sFRPs), which influence the activation of receptor [47]. Furthermore, β‐catenin interacts with a range of transcriptional coactivators and chromatin‐modifying enzymes, complicating efforts to selectively inhibit oncogenic signaling while preserving its physiological functions [43].

Recent advancements have focused on developing agents to modulate the Wnt/β‐catenin pathway, including inhibitors targeting Wnt ligands, monoclonal antibodies (mAbs) against Wnt receptors, and small interfering RNAs designed to silence the expression of key pathway components [48, 49, 50]. These strategies hold promise for disrupting oncogenic signaling while minimizing off‐target effects, although further preclinical and clinical studies are needed to optimize their efficacy and safety profiles.

#### 2.2.6 NOTCH Pathway

The NOTCH signaling is integral to cellular communication and gene expression, mediated through receptor–ligand interactions. Upon ligand binding, NOTCH receptors (1–4) undergo proteolytic cleavage, releasing the intracellular domain (NICD), which translocates to the nucleus and activates downstream transcription factors [51].

In PDAC, aberrant NOTCH signaling accelerates tumor aggressiveness. NOTCH receptor activation induces genes associated with cell cycle progression and inhibits apoptotic pathways. Additionally, NOTCH signaling promotes EMT, where epithelial cells lose adhesion and acquire mesenchymal traits, facilitating metastasis [52]. Meanwhile, NOTCH signaling sustains the stemness of cancer cells, maintaining the self‐renewal capacity. This contributes to tumor recurrence and long‐term growth. Consequently, NOTCH signaling not only drives tumor progression but also confers therapeutic resistance in PDAC [53, 54, 55]. For example, Du et al. [54] found that, blocking NOTCH signaling could increase the sensitivity of PC cells to gemcitabine (GEM) by triggering the intrinsic apoptotic pathway. Furthermore, as glioma amplified sequence 41 (GAS41) is a new regulator of NOTCH signaling via modulating H2A.Z deposition, evidence indicated that GAS41 interacts with H2A.Z.2 to activate NOTCH‐1 signaling and its downstream, which promote PC stemness and resistance to GEM [56, 57].

Targeting the NOTCH pathway is challenging due to the potential toxicity to normal tissues, as NOTCH signaling is essential for adult stem cell homeostasis. Additionally, tumor heterogeneity, including variations in receptor expression and ligand presence, complicates the development of selective therapies. Understanding the distinct roles of NOTCH receptors and ligands in PDAC could enable more selective and effective therapeutic strategies.

## 3 Systemic Treatments for PDAC

PDAC remains one of the most lethal malignancies, necessitating the comprehensive therapeutic approach to improve patient survival outcomes. Although conventional chemotherapy remains the mainstay of systemic treatment, recent advancements in various strategies highlight the importance of novel interventions to overcome therapeutic resistance and address the profound heterogeneity inherent to PDAC.

### 3.1 Chemotherapy

Chemotherapy continues to serve as the cornerstone in PDAC management, with distinct roles in neoadjuvant, adjuvant, and advanced disease settings. Neoadjuvant chemotherapy aims to reduce tumor stage and facilitate surgery, while adjuvant chemotherapy is critical for preventing recurrence. Significant progress in chemotherapy regimens, such as FOLFIRINOX and GEM‐based combinations, has shown promising results in different settings (Table 1).

**TABLE 1:** Milestone studies of chemotherapy in pancreatic ductal adenocarcinoma.

Trial	Phase	Identifier	Setting	Treatment	Outcomes	References
Prep‐02/JSAP‐05	III	UMIN000009634	Neoadjuvant	GS vs. upfront surgery	mOS: 36.7 vs. 26.6 months; *p* = 0.015	[58]
SWOG S1505	II	NCT02562716	Neoadjuvant	mFOLFIRINOX vs. GEM–NabP	mOS: 23.2 vs. 23.6 months	[59]
ESPAC‐5	II	ISRCTN89500674	Neoadjuvant	Immediate surgery vs. GEM + Cap vs. FOLFIRINOX vs. Cap‐based CRT	1‐year OS rate: 2–39 vs. 78 vs. 84 vs. 60%	[60]
CSGO‐HBP‐015	II	UMIN000021484	Neoadjuvant	GEM–NabP vs. GS	mPFS: 14.0 vs. 9.0 months; *p* = 0.048	[61]
PREOPANC‐1	III	EudraCT: 2012‐003181‐40	Neoadjuvant	Upfront surgery vs. neoadjuvant CRT	mOS: 15.7 vs. 14.3 months; *p* = 0.025	[62]
PREOPANC‐2	III	EudraCT: 2017‐002036‐17	Neoadjuvant	FOLFIRINOX vs.CRT	mOS: 21.9 vs. 21.3 months; *p* = 0.28	[63]
Alliance A021501	II	NCT02839343	Neoadjuvant	mFOLFIRINOX vs. mFOLFIRINOX + hypofractionated RT	mOS: 29.8 vs. 17.1 months	[64]
ESPAC‐1	—	—	Adjuvant	CRT alone vs. chemotherapy alone vs. CRT and chemotherapy vs. observation	mOS: 13.9 vs. 21.6 vs. 19.9 vs. 16.9 months	[65]
CONKO‐001	III	ISRCTN34802808	Adjuvant	GEM vs. observation	mDFS: 13.4 vs. 6.7 months; *p* < 0.001	[66]
CONKO‐005	III	EudraCT2007‐003813‐15	Adjuvant	GEM + erlotinib vs. GEM	mDFS: 11.4 vs. 11.4 months	[67]
ESPAC‐3	III	NCT00058201	Adjuvant	Fluorouracil vs. GEM	mOS: 23.0 vs. 23.6 months; *p* = 0.39	[68]
ESPAC‐4	III	EudraCT: 2007‐004299‐38 ISRCTN96397434	Adjuvant	GEM + Cap vs. GEM	mOS: 28.0 vs. 25.5 months; *p* = 0.032	[69]
JASPAC‐01	III	UMIN000000655	Adjuvant	S‐1 vs. GEM	mOS: 46.5 vs. 25.5 months; *p* < 0.05	[70]
PRODIGE 24/CCTG PA6	III	NCT01526135	Adjuvant	mFOLFIRINOX vs. GEM	mOS: 54.4 vs. 35.0 months; *p* = 0.003	[71]
APACT	III	NCT01964430	Adjuvant	GEM–NabP vs. GEM	mDFS: independent review: 19.4 vs. 18.8 months; *p* = 0.18; investigator‐assessed: 16.6 vs. 13.7 months; *p* = 0.02	[72]
—	III	—	Advanced	GEM + Cap vs. GEM	mPFS: 5.3 vs. 3.8; *p* = 0.004	[73]
PRODIGE	III	NCT00112658	Advanced	FOLFIRINOX vs. GEM	mOS: 11.1 vs. 6.8; *p* < 0.001 mPFS: 6.4 vs. 3.3; *p* < 0.001	[74]
MPACT	III	NCT00844649	Advanced	GEM–NabP vs. GEM	mOS: 8.5 vs. 6.7; *p* < 0.001	[75]
NAPOLI‐1	III	NCT01494506	Advanced	nal‐IRI + 5‐FU vs. nal‐IRI monotherapy vs. 5‐FU	mOS: 6.1 vs. 4.9 vs. 4.2	[76]
NAPOLI‐3	III	NCT04083235	Advanced	NALIRIFOX vs. GEM–NabP	mOS: 11.1 vs. 9.2; *p* = 0.036	[77]

*Abbreviations*: GS, gemcitabine + S‐1; mOS, median overall survival; GEM, gemcitabine; Cap, capecitabine; GEM–NabP, gemcitabine + nab‐Paclitaxel; mPFS, median progression‐free survival; CRT, chemoradiotherapy; RT, radiotherapy; mDFS, median disease‐free survival; nal‐IRI, nanoliposomal irinotecan; 5‐FU, 5‐fluorouracil; NALIRIFOX, liposomal irinotecan, oxaliplatin, leucovorin, and fluorouracil

#### 3.1.1 Neoadjuvant Chemotherapy

Neoadjuvant chemotherapy has demonstrated significant efficacy across a spectrum of malignancies, optimizing treatment adherence and improving survival outcomes, particularly in borderline resectable disease. In recent years, its application in PDAC has led to substantial progress.

Prep‐02/JSAP‐05 trial, the first phase III study of neoadjuvant therapy in PDAC, compared neoadjuvant GEM plus S‐1 (GS) followed by surgery and adjuvant S‐1. While surgical outcomes, including R0 resection rates, morbidity, and mortality, were comparable, median overall survival (mOS) was significantly improved in the neoadjuvant group (36.7 vs. 26.6 months) [58]. However, these findings did not change clinical practice, as the results were inconsistent with those of the JASPAC‐01 trial, which reported a mOS of 46.5 months with adjuvant S‐1 and 25.5 months with GEM. Similarly, the SWOG S1505 trial, comparing perioperative mFOLFIRINOX and GEM/nab‐Paclitaxel (GEM–NabP), showed no significant difference in 2‐year OS rate (47 vs. 48%) or mOS (23.2 vs. 23.6 months) [59]. And the NEONAX trial, comparing perioperative GEM–NabP with adjuvant GEM–NabP, failed to meet its primary endpoint of a disease‐free survival (DFS) rate >55% at 18 months [78].

Regardless of these uninspiring results, certain clinical trials have reported promising results. For instance, the ESPAC‐5 trial demonstrated superior survival benefits with neoadjuvant regimens, including GEM plus capecitabine or FOLFIRINOX, compared with immediate surgery [60]. Additionally, recent findings from the CSGO‐HBP‐015 study revealed that GEM–NabP and GS are effective neoadjuvant treatment for PDAC, with the GEM–NabP arm showing a favorable progression‐free survival (PFS) [61].

Although neoadjuvant chemotherapy has shown limited efficacy in PDAC, studies on neoadjuvant chemoradiotherapy (CRT) have yielded promising results. In the PREOPANC‐1 study, 246 patients were randomized to upfront surgery with adjuvant GEM or neoadjuvant CRT with GEM followed by surgery and adjuvant GEM. Long‐term follow‐up showed improved mOS in the neoadjuvant group (15.7 vs. 14.3 months), with 5‐year OS rate of 20.5 and 6.5%, respectively. Subgroup analysis indicated a significant OS improvement with neoadjuvant CRT (17.6 vs. 13.2 months, *p* = 0.029) [62]. In addition, the phase III PREOPANC‐2 study compared neoadjuvant FOLFIRINOX with GEM‐based neoadjuvant CRT in borderline resectable PDAC. Although preliminary results showed no significant difference in mOS (21.9 months for FOLFIRINOX vs. 21.3 months for CRT, *p* = 0.28) and surgical resection rate (77% for FOLFIRINOX vs. 75% for CRT, *p* = 0.69), the proportion of postoperative lymph node‐negative (N0) patients was significantly higher in the CRT group (58 vs. 47%, *p* < 0.01). Both regimens showed comparable efficacy and safety profiles, while grade 3–4 diarrhea and neutropenic fever were less frequent in the CRT group [63]. It is worth noting that the total dose of combined radiotherapy was only 36 Gy (15 fractions of 2.4 Gy), which was lower than standard neoadjuvant radiotherapy dose. Besides, in the Alliance A021501 trial, candidates were randomized to neoadjuvant mFOLFIRINOX with or without hypofractionated radiation, followed by mFOLFOX6 as adjuvant therapy. The mOS of CRT group was inferior than that of chemotherapy group (17.1 vs. 29.8 months) [64]. Meanwhile, a meta‐analysis of several large randomized trials demonstrated that neoadjuvant therapy did not significantly improve OS or DFS in resectable PDAC patients [79].

Neoadjuvant treatment in PDAC leads to tumor shrinkage and increases the opportunity of R0 resection, directly correlating with improved survival. However, there still remains controversial. How to optimize neoadjuvant strategies is worth further exploration. Incorporating tailored drug regimens, such as FOLFIRINOX, alongside precision dose adjustments, represents a promising approach to maximizing therapeutic efficacy while minimizing AEs.

#### 3.1.2 Adjuvant Chemotherapy

Adjuvant chemotherapy has gained substantial attention in the management of PDAC. Clinical evidence demonstrates that adjuvant chemotherapy significantly reduces recurrence rates and improves OS in PDAC patients.

The ESPAC‐1 is a pivotal trial, clarifying the role of chemotherapy as adjuvant therapy for PDAC [65]. The trial randomized resected PDAC patients into four groups: chemoradiotherapy alone (20 Gy over a 2‐week period plus fluorouracil), chemotherapy alone (fluorouracil), both chemoradiotherapy and chemotherapy, and observation. Results indicated that chemotherapy alone significantly improved survival compared with observation (20.1 vs. 15.5 months, *p* = 0.009), whereas chemoradiotherapy increased mortality risk (15.9 vs. 17.9 months, *p* = 0.05). In parallel, the CONKO‐001 trial, involving R0 resected PDAC patients, evaluated GEM as a postoperative adjuvant treatment [66]. GEM significantly improved DFS (13.4 vs. 6.7 months, *p* < 0.001) and OS (5‐year OS: 20.7 vs. 10.4%, *p* = 0.01), compared with observation. But in the subsequent CONKO‐005 trail, the results failed to show the benefit of GEM combined with erlotinib in adjuvant setting [67].

Given that both 5‐fluorouracil (5‐FU) and GEM could prolong survival as adjuvant treatment, direct comparisons are performed. The ESPAC‐3 phase III trial found there was no significant difference in mOS between the two agents (23.6 vs. 23.0 months, *p* = 0.39). Fourteen percent of candidates receiving 5‐FU had treatment‐related serious AEs, compared with 7.5% of those receiving GEM (*p* < 0.001) [68]. To explore more effective regimens, the ESPAC‐4 trial assessed the combination of GEM and capecitabine. The combination regimen resulted in a mOS of 28.0 months, compared with 25.5 months for GEM alone (*p* = 0.032), thus providing a more effective option [69]. Although GEM has been a cornerstone of adjuvant treatment, its efficacy has been challenged by the GEST study, which found S‐1 was noninferior to GEM in advanced PDAC [80]. The JASPAC‐01 trial further demonstrated that S‐1 significantly improved OS (46.5 vs. 25.5 months for GEM, *p* < 0.05), with a 5‐year OS rate of 44.1% [70]. Notably, patients treated with S‐1 had earlier‐stage disease and more D2 resections. And this study was conducted in an Asian cohort, highlighting the need for further investigation in non‐Asian populations.

Currently, there are several trials evaluating different regimens in adjuvant setting. The PRODIGE 24/CCTG PA6 trial, which enrolled 493 resected PDAC patients, found that mFOLFIRINOX significantly improved OS, compared with GEM (54.4 vs. 35.0 months, *p* = 0.003). Subgroup analyses consistently favored mFOLFIRINOX over GEM. However, due to the high toxicity of mFOLFIRINOX regimen, more than half of patients received granulocyte colony‐stimulating factor (G‐CSF), and a significant proportion necessitated dose modifications to enhance treatment tolerability [71]. Nevertheless, this trial highlights the therapeutic potential of mFOLFIRINOX as an effective adjuvant therapy.

The APACT phase III trial evaluated the efficacy of GEM–NabP versus GEM alone in patients with resected PDAC. While the independent review demonstrated no statistically significant difference in mDFS between the two arms (19.4 vs. 18.8 months, p = 0.18), investigator‐assessed DFS favored the combination regimen (16.6 vs. 13.7 months, *p* = 0.02), and OS was significantly prolonged in the GEM–NabP group (40.5 vs. 36.2 months, *p* = 0.045) [72]. Despite the observed OS benefit, the trial failed to meet its primary endpoint of independently assessed DFS, leading to restricted endorsement of GEM–NabP in current clinical guidelines.

#### 3.1.3 Chemotherapy for Advanced PDAC

In 1997, Burris et al. [81] demonstrated the superiority of GEM monotherapy over 5‐FU in advanced PDAC. Despite this breakthrough, progress in chemotherapy development remained limited for nearly two decades until the emergence of results from subsequent landmark phase III trials. Among these, the efficacy of combining GEM with capecitabine was evaluated against GEM monotherapy in advanced PDAC. While no significant difference was observed in mOS, a statistically significant improvement in PFS was achieved in the combination arm (5.3 vs. 3.8 months, *p* = 0.004) [73]. Concurrently, the PRODIGE trial established the substantial superiority of the FOLFIRINOX regimen over GEM monotherapy in advanced PDAC, with a mOS of 11.1 months and mPFS of 6.4 months in the FOLFIRINOX arm compared with 6.8 and 3.3 months in the GEM arm, respectively. However, it is worth note that the FOLFIRINOX regimen is associated with increased toxicity and inferior quality of life, which constrains its application in clinical practice [74]. Subsequently, the phase III MPACT trial compared the combination of NabP and GEM with GEM monotherapy in advanced PDAC. The combined regimen provided a significantly improved mOS (8.5 vs. 6.7 months, *p* < 0.001) [75]. These trials collectively represent critical milestones in the evolution of systemic therapy for advanced PDAC, highlighting the trade‐offs between efficacy, toxicity, and clinical feasibility.

Advances in nanotechnology have enabled the development of nanoparticles with several advantageous characteristics for drug delivery, including the potential to overcome the tumor interstitial barrier and enhance therapeutic efficacy [82]. Among these innovations, nanoliposomal irinotecan has emerged as a prominent example. Its efficacy was demonstrated in a pivotal trial involving advanced PDAC patients previously treated with GEM‐based regimens. The study reported a significant improvement in mOS for patients receiving nanoliposomal irinotecan combined with 5‐FU/leucovorin, compared with those receiving 5‐FU/leucovorin alone (6.2 vs. 4.2 months, *p* = 0.042) [76]. More recently, the NAPOLI‐3 trial revealed compelling evidence supporting the use of NALIRIFOX (liposomal irinotecan, oxaliplatin, leucovorin, and fluorouracil) as first‐line therapy for advanced PDAC. The study demonstrated a mOS of 11.1 months in the NALIRIFOX arm, significantly surpassing the 9.2 months achieved with GEM–NabP regimen (*p* = 0.036) [77]. These findings have led to the approval of NALIRIFOX as a standard first‐line treatment for advanced PDAC. Furthermore, the combination of GEM and NabP with cisplatin has shown promising efficacy in advanced PDAC. A phase Ib/II trial reported an overall response rate of 71% and a disease control rate (DCR) of 88% with this combined strategy [83]. However, the primary concern regarding combined regimens is toxicity, while devising strategies to improve the efficacy of combined therapy warrants consideration.

#### 3.1.4 Outlooks

Emerging evidence suggests that the efficacy of chemotherapy varies across different subtypes of PDAC. For example, in the COMPASS trial, investigators conducted whole genome sequencing and RNA sequencing (RNA‐seq) on candidates with advanced PDAC to identify predictive mutational and transcriptional signatures. Treatment with FOLFIRINOX resulted in a PFS rate of 60% among patients with basal‐like tumors, compared with only 15% in those with classical PDAC (*p* = 0.0002) [84, 85]. These findings have been corroborated by subsequent studies, further validating the association between molecular subtypes and therapeutic outcomes [86]. Moreover, retrospective analyses revealed that patients harboring BRCA1/2 mutations exhibited a significantly higher response rate to platinum‐based therapies compared with their nonmutated counterparts [87, 88]. Collectively, these findings highlight the role of prospective genomic profiling in guiding treatment.

Chemoresistance remains a persistent challenge. Accumulating evidence indicates that multidrug resistance (MDR) proteins play a pivotal role in mediating chemoresistance through several mechanisms, including the activation of antiapoptotic pathways, enhanced intracellular drug efflux, and the reduction of drug concentrations within tumor cells [89]. Notably, resistance to GEM in PC cells has been closely associated with the overexpression of MDR proteins, highlighting the necessity of targeting these proteins to restore chemotherapeutic efficacy [90, 91]. Beyond MDR proteins, dysregulation of apoptosis signaling pathways, particularly the upregulation of Bcl‐2 family proteins, has been implicated as a critical driver of chemoresistance [92, 93]. Downregulating their expression could enhance GEM‐induced apoptosis, offering a potential strategy to overcome resistance. Furthermore, emerging research has underscored the contribution of metabolic reprogramming to chemoresistance [94]. GEM‐resistant cells exhibit increased mitochondrial density and aberrant expression of mitochondrial fission proteins, which promotes oxidative phosphorylation and upregulates antiapoptotic proteins [95]. These findings collectively suggest that targeting mitochondrial dynamics and metabolism represents a promising avenue for circumventing chemoresistance.

TME is another factor contributing to chemoresistance. Pancreatic tumors are characterized by extensive fibrosis, primarily composed of collagen and ECM components, which markedly increase tumor stiffness and create a physical barrier that impedes drug penetration and distribution [96]. Beyond its structural role, fibrosis activates prosurvival signaling pathways, such as TGF‐β, further exacerbating chemotherapy resistance [97]. Additionally, the abundant of fibrotic stroma compresses blood vessels, fostering a hypovascular and hypoxic microenvironment that severely hampers the delivery and uptake of drugs, thus contributing to the poor response of PDAC to chemotherapy. The disorganized vasculature further compounds the issue by causing uneven drug distribution, leading to subtherapeutic drug concentrations in certain regions and increasing the risk of resistance [98, 99]. To address these barriers, significant efforts have been directed toward developing strategies to disrupt tumor fibrosis and normalize vascular abnormalities. Although preclinical studies have demonstrated promising results, overcoming fibrosis and vascular defects in PDAC remains a significant challenge. Future research should prioritize the identification of optimal combination therapies capable of reversing fibrosis and reconstructing functional vasculature, which could significantly enhance therapeutic outcomes in PDAC.

As for the safety profile of combined chemotherapy, tailoring therapeutic agents and dosages according to the patient's overall health status is essential to minimize the incidence and severity of AEs. To further enhance safety, regular monitoring of hematological parameters and organ function is critical, enabling timely adjustments to chemotherapy regimens to mitigate potential toxicities. Additionally, the implementation of supportive care is also important. For instance, the administration of growth factors in patients at high risk for febrile neutropenia can effectively alleviate chemotherapy‐induced cytopenias [100]. Equally important is the management of potential drug interactions. A comprehensive assessment of concomitant medications that may impact the efficacy of chemotherapy should be performed prior to treatments, aiming to avoid the use of drugs that interact adversely with chemotherapeutic agents [101]. In summary, optimizing the safety of combined chemotherapy requires a multifaceted approach, incorporating personalized dosing, vigilant monitoring, supportive care, and careful management of drug interactions.

### 3.2 Targeted Therapy

PDAC manifests a diverse array of pathways, encompassing key regulators like EGFR, RAS, poly‐ADP ribose polymerase (PARP), vascular endothelial‐derived growth factor (VEGF) and its receptor (VEGFR). These pathways exhibit significant associations with pivotal cellular processes in pancreatic malignancy. Substantial preclinical studies have validated their efficacy in PC, thereby facilitating the advancement of related clinical investigations (Table 2).

**TABLE 2:** Preclinical antitumor effect of agents in pancreatic cancer.

Target	Agents	Potential antitumor mechanism	References
EGFR	Erlotinib	Arrest cell cycle, induce cell apoptosis and suppress capillary formation of endothelium	[102]
	Gefitinib	Arrest cell cycle	[103]
	Afatinib	Distribute cell cycle and block the EGF‐induced phosphorylation of tyrosine	[104]
	Cetuximab	Inhibit EGFR‐dependent proliferation and survival	[105]
	Nimotuzumab	Block the overexpressing of EGFR	[106]
RAS	Sotorasib	Selectively inhibit KRAS G12C, induce apoptosis and alter TME by recruiting stromal cells, such as fibroblasts and macrophages	[107]
	GDC‐6036	Inhibit KRAS G12C alkylation and MAPK pathway	[108]
	MRTX1133	Inhibit KRAS G12D and ERK1/2 phosphorylation	[109]
	RT11‐i	Target the activated GTP‐bound form of RAS mutant and inhibit the downstream of RAS signaling pathway	[110]
	RT22‐ep59	Target the intracellularly activated GTP‐bound form of KRAS mutant	[111]
	BI‐2865	Bind to the inactive state of KRAS	[112]
	RMC‐7977	Highly inhibit the active GTP‐bound forms of RAS, induce apoptosis and arrest proliferation	[113]
	RMC‐6236	Inhibit the GTP‐bound state of multiple RAS variants	[114]
PARP	Venadaparib	Inhibit PARP‐1 and ‐2 enzymes	[115]
	Saruparib	Selectively inhibit PARP‐1	[116]
Multitargets	Sorafenib	Induce apoptosis, inhibit angiogenesis and downregulate ERK pathway	[117]
	Sunitinib	Attenuate radiation‐induced phosphorylation of AKT and ERK	[118]
MEK	Trametinib	The G12C mutant KRAS activates primarily the MEK/ERK pathway, which contributes to the efficacy of trametinib in KRAS–G12C mutant cell.	[119]
RAF	LY3009120	Its combination with ERK inhibitor has highly synergistic antitumor effect in KRAS mutant PDAC cell lines, due to loss of ERK signaling, FOSL1, and MYC; shutdown of the MYC transcriptome; and induction of mesenchymal‐to‐epithelial transition.	[26]
IGF‐IR	NVP‐AEW541	Diminish the activation of IGF‐IR, IRS‐1, ERK, AKT, and STAT3 and reduce the vascularization	[120]
	Ganitumab	Inhibit IGF‐1‐induced AKT phosphorylation	[121]
NOTCH 2/3	Tarextumab	Inhibit human NOTCH 2/3 reporter activity and reverse EMT	[122]
γ‐Secretase	MRK‐003	Inhibit NOTCH pathway and block epithelial proliferation	[123]
TGF‐βR	Vactosertib	Inhibit the TGF‐β/SMAD2 pathway and ECM component production	[124]
CDK4/6	Palbociclib	Induce G1‐phase arrest, reduce cell viability and promote an epithelial phenotype	[125]
	Palbociclib	Its combination with nab‐Paclitaxel shows promising efficacy, but the mechanism is not well elucidated.	[126]
	Palbociclib	Its combination with an ERK inhibitor (ERKi) synergistically suppresses the growth of tumor cell lines by counteracting the compensatory upregulation of ERK signals induced by CDK4/6 inhibition.	[127]
	Abemaciclib	Its combination with a HDAC inhibitor panobinostat induce synergistic apoptosis.	[128]

*Abbreviations*: EGFR, epidermal growth factor receptor; EGF, epidermal growth factor; TME, tumor microenvironment; MAPK, mitogen‐activated protein kinase; GTP, guanosine triphosphate; PARP, poly‐ADP ribose polymerase; PDAC, pancreatic ductal adenocarcinoma; IGF‐IR, insulin‐like growth factor‐I receptor; IRS, insulin receptor substrate; VEGF, vascular endothelial‐derived growth factor; EMT, epithelial‐to‐mesenchymal transition; TGF‐βR, transforming growth factor‐β receptor; ECM, extracellular matrix; CDK4/6, cyclin‐dependent kinase 4/6

#### 3.2.1 Agents Targeting EGFR Pathway

The landscape of agents targeting the EGFR pathway has undergone rapid evolution, predominantly featuring mAbs and tyrosine kinase inhibitors (TKIs). Through competitive binding with ATP, EGFR‐TKIs exert reversible inhibition on the EGFR tyrosine kinase domain [129].

Erlotinib, the first‐generation EGFR‐TKI, has exhibited robust antitumor activity in vitro and in vivo, primarily through mechanisms involving cell cycle arrest and induction of apoptosis [102]. Its combination with GEM showed significant improvements in OS for advanced PDAC [130]. However, its therapeutic efficacy is constrained by challenges such as drug antagonism and the dense fibrotic TME, which severely limits drug penetration. To address this, Tang et al. [131] developed an innovative dual stimuli‐responsive delivery system for the codelivery of GEM and erlotinib, which enhanced antitumor effects by reshaping the TME and inhibiting cell proliferation and migration. Gefitinib, another first‐generation EGFR‐TKI, has been shown to induce G0/G1 cell cycle arrest by upregulating the CDK inhibitor p27Kip1, thereby effectively inhibiting tumor cell proliferation [103]. Although its combination with GEM and radiotherapy showed acceptable toxicity in a phase I trial, this regimen failed to achieve sufficient clinical efficacy to warrant further investigation [132]. Similarly, subsequent studies exploring gefitinib in combination with chemotherapy in advanced PDAC did not yield positive outcomes, highlighting the limited utility of this agent in this context [133, 134]. Afatinib, an irreversible ErbB family inhibitor, was firstly approved for EGFR mutation‐positive non‐small‐cell lung cancer (NSCLC) [135]. It has shown promise in PC by inhibiting EGFR and downstream signaling pathways (e.g., MAPK, AKT) [104]. However, its combination with GEM did not significantly improve survival in PDAC [136]. Despite this, certain studies suggested its potential benefits in advanced PDAC patients with KRAS wild‐type tumors harboring NRG1 gene fusions, which warrants further investigation [137].

As for osimertinib, the third‐generation of EGFR‐TKIs, its efficacy in PDAC is very limited. Previous study identified osimertinib‐resistant EGFR triple mutations (Del19/T790M/C797S or L858R/T790M/C797S). In response to this challenge, researchers have developed bioavailable agents specifically designed to target these resistant mutations. For instance, Du et al. [138] demonstrated that HJM‐561, an oral EGFR proteolysis‐targeting chimera, exhibits significant antitumor activity in animal models driven by EGFR triple mutations that are resistant to osimertinib. Similarly, Kashima et al. [139] reported that CH7233163, a novel EGFR inhibitor, shows potential in overcoming resistance conferred by EGFR‐Del19/T790M/C797S mutations. Combined therapy is also a strategy to overcome the resistance of osimertinib. Doxazosin is a classic quinazoline‐based alpha 1‐adrenoceptor antagonist. By inducing autophagy, its combination with osimertinib elicited synergic antitumor effect on NSCLC and PC cells [140]. Collectively, these findings highlight the critical need to develop innovative strategies to overcome EGFR‐TKI resistance, which remains a pivotal focus in the development of next‐generation EGFR‐TKIs.

mAbs have emerged as a promising treatment for PC due to their specific target and manageable toxicity. Cetuximab, an anti‐EGFR mAb, is currently approved for the treatment of advanced head and neck squamous cell carcinoma and metastatic colorectal cancer (CRC). Preclinical studies have demonstrated that cetuximab effectively disrupts EGFR‐mediated signaling pathways, leading to significant inhibition of proliferation and survival in PC cells both in vitro and in vivo [105]. However, these promising results were not born out in clinical studies [141, 142, 143]. Several factors may contribute to this. CRC patients with KRAS or NRAS mutations are ineligible for cetuximab treatment [144], and over 90% of PDAC patients harbor KRAS mutations [145]. Hence, the modest efficacy of cetuximab in PDAC is not unexpected. To address this challenge, recent studies have investigated alternative approaches. For example, cetuximab‐resistant clones exhibit heightened reliance on HER family receptors, prompting evaluation of HER inhibitors in cetuximab‐resistant cells. Remarkably, HER inhibitors attenuated cell proliferation in such clones by deactivating AKT and MAPK signaling pathways, demonstrating therapeutic promise in vivo [146, 147]. However, a phase I/II trial failed to observe objective responses in advanced PDAC patients treated with cetuximab plus trastuzumab [148].

Nimotuzumab, a humanized mAb targeting EGFR, exerts its antitumor effects by inhibiting EGFR overexpression in PC cells [106]. This agent has demonstrated therapeutic potential when combined with GEM for advanced PDAC [149]. In a phase II clinical trial, patients who relapsed after first‐line chemotherapy were administered nimotuzumab as a salvage therapy, resulting in a mOS of 18.1 weeks [150]. These encouraging findings prompted further investigation into the combination of nimotuzumab and GEM for PDAC. Positive outcomes demonstrated that this combination significantly enhanced 1‐year OS and PFS rates, particularly in advanced PDAC patients with wild‐type KRAS [151]. Recently, the findings from the NOTABLE study, a prospective, randomized phase III trial evaluating the efficacy of nimotuzumab combined with GEM versus GEM monotherapy in advanced PDAC patients with KRAS wild‐type, demonstrated a statistically significant improvement in both mOS (10.9 vs. 8.5 months, *p* = 0.024) and mPFS (4.2 vs. 3.6 months, *p* = 0.013). In terms of safety, comparable incidence of AEs was observed in two groups [152]. Consequently, nimotuzumab combined with GEM gained approval for this patient population by the Chinese National Medical Products Administration.

The resistance to EGFR inhibitors has garnered significant attention. Various factors contribute to this, including the T790M mutation, which has been addressed through the successful development of third‐generation EGFR‐TKIs [153]. However, clinical practice revealed the emergence of resistance to third‐generation EGFR‐TKIs, thereby driving the development of fourth‐generation alternatives. BLU‐945, for instance, represents a reversible inhibitor sparing wild‐type EGFR and targeting EGFR with T790M or T790M/C797S mutations. Eno et al. [154] demonstrated that BLU‐945 exhibits significant kinome selectivity. In animal models resistant to osimertinib, BLU‐945 effectively suppressed tumor progression while maintaining acceptable safety profile, thereby warranting further clinical evaluation. Presently, BLU‐945 is undergoing assessment in a phase I/II clinical trial for the treatment of EGFR‐driven NSCLC resistant to conventional therapies (NCT04862780). Mobocertinib, a pioneering EGFR‐TKI, has been developed specifically for NSCLC characterized by EGFR exon 20 insertions (EGFRex20ins). Preclinical investigations have confirmed the efficacy of mobocertinib in various EGFRex20ins‐driven cell lines [155]. Subsequent phase I/II clinical trial indicated that mobocertinib is associated with improved survival outcomes in patients with previously treated EGFRex20ins‐positive NSCLC [156]. Consequently, mobocertinib received accelerated approval from the FDA for this indication. Prospective evaluation of these innovative agents in PDAC is eagerly anticipated.

The activation of bypass signaling pathways, such as MET amplification and HER2 upregulation, represents a key mechanism underlying resistance to EGFR‐TKIs [157]. To address this challenge, combination therapies involving targeted agents that simultaneously inhibit these alternative pathways have emerged as a promising strategy. For instance, Blasco et al. [158] identified that the expression of both EGFR and c‐RAF is essential for the proliferation of PC cells, highlighting the potential therapeutic value of dual inhibition. Utilizing EGFR‐TKIs and c‐RAF‐specific shRNAs, the proliferation of PC cell was completely suppressed. Furthermore, vivo studies demonstrated that combined inhibition of EGFR and c‐RAF significantly impeded tumor progression in models harboring KRAS and TP53 mutation, providing robust preclinical evidence to support the development of targeted therapies for PC [158]. Additionally, EMT has been implicated as a critical contributor to TKI resistance [159, 160]. Given the dynamic and multifaceted nature of EMT, further investigation is warranted to elucidate its underlying mechanisms and identify effective therapeutic strategies. Notably, AXL kinase activation has been proposed as a mediator of EMT in this context, suggesting that targeting the AXL pathway may represent a viable approach to overcoming EGFR‐TKI resistance [161].

In parallel, nanobodies (Nbs), especially those targeting EGFR, have garnered considerable attention as a promising avenue in targeted therapy. While anti‐EGFR Nbs exhibit significant efficacy against EGFR‐overexpressing solid tumors, critical challenges persist in optimizing their binding affinity and minimizing immunogenicity, which are essential for their clinical translation and therapeutic success [162, 163].

#### 3.2.2 Agents Targeting RAS Pathway

KRAS mutation serves as a pivotal driver of tumorigenesis and is closely linked to poor prognosis in PDAC. This oncogenic mutation modulates diverse biological processes in PC, such as macropinocytosis and autophagy, while concurrently activating downstream signaling pathways, including AKT and mTOR, which collectively promote tumor progression and metastasis in KRAS‐mutated PDAC [164, 165]. In light of these findings, significant efforts have been directed toward developing therapeutic inhibitors targeting KRAS and its downstream effector. A prior investigation delineated the involvement of KRAS G12C in both its active GTP‐bound and inactive GDP‐bound states, thus laying a foundation for the development of targeted therapy [107].

Sotorasib is a member of the RAS GTPase family inhibitors. Lee et al. [166] found that in a patient‐derived xenograft (PDX) model harboring KRAS G12C‐mutation, sotorasib effectively inhibited KRAS G12C both in vitro and in vivo, while also modulating the TME. Clinically, sotorasib has been evaluated in patients with previously treated KRAS p.G12C‐mutated NSCLC, achieving an objective response rate (ORR) of 37.1%, with mPFS and mOS of 6.8 and 12.5 months, respectively [167]. Further, the CodeBreaK100 study, which included 38 patients with advanced PDAC, reported mPFS and mOS of 4.0 and 6.9 months, respectively. Common AEs were limited to gastrointestinal symptoms and pyrexia, with no grade 4 or 5 treatment‐related AEs observed [168]. Similarly, adagrasib, another KRAS G12C inhibitor, has shown notable antitumor efficacy in advanced solid tumors harboring this mutation. In the phase II KRYSTAL‐1 study, which enrolled 21 advanced PDAC patients with KRAS G12C‐mutant, an ORR of 33.3% was achieved, along with mPFS and mOS of 5.4 and 8.0 months, respectively [169]. These encouraging results have led to the initiation of a phase Ib trial (NCT05634525) to further assess the safety and efficacy of adagrasib in advanced PDAC patients with KRAS G12C mutation (NCT05634525).

Divarasib, also known as GDC‐6036, is a covalent KRAS G12C inhibitor [108]. A phase I study assessed the safety and efficacy of divarasib monotherapy in patients with advanced solid tumors harboring KRAS G12C mutation. Among patients with advanced PDAC, three achieved partial response (PR) and four had stable disease (SD). Across all cancer types, most AEs were low‐grade, including nausea, diarrhea, and vomiting [170]. Glecirasib is a highly selective inhibitor of KRAS G12C. Ongoing phase I/II trials (NCT05009329 and NCT05002270) are evaluating its safety and efficacy in solid tumors harboring KRAS G12C mutation. Preliminary findings indicated that among 28 participants with PDAC, 13 achieved a confirmed PR, resulting in a confirmed ORR of 46.4% and a DCR of 96.4%. The mPFS was 5.5 months. The most common AEs included hematological toxicity and liver dysfunction. These results underscore the considerable antitumor activity of glecirasib in patients with KRAS G12C‐mutated PDAC [171]. Furthermore, several novel agents, including KRAS G12C inhibitor JDQ443 (NCT04699188) and KRAS G12D inhibitor MRTX1133 (NCT05737706), are currently evaluating in clinic [109, 172, 173], with anticipation for positive outcomes.

Concurrently, the development of antibodies targeting KRAS, such as RT11‐i antibody, is underway. The study performed by Kang et al. [110] demonstrated that RT11‐i, by targeting the activated GTP‐bound form of RAS mutants and inhibiting the downstream of RAS signaling pathway, synergistically enhanced the antitumor activity of GEM in PC cells. Moreover, this combined treatment preserved endothelial barrier integrity, thus impeding metastasis [110]. RT22‐ep59, another antibody targeting KRAS, sensitizes PC cells to GEM, particularly those exhibiting high epithelial cell adhesion molecule (EpCAM) expression [111]. These findings underscore the efficacy of KRAS‐targeting antibodies when combined with GEM in PDAC with KRAS mutation.

Despite inspiring advancements in the development of KRAS inhibitors, obstacles persist in optimizing their effectiveness. Presently, KRAS inhibitors predominantly target the G12C mutation, which is not frequent in PDAC [174]. Given that G12D and G12V mutations are more prevalent in PDAC, agents tailored to these variants or pan‐KRAS inhibitors hold considerable promise. Fortunately, the development of pan‐KRAS inhibitors has made significant strides. Kim et al. [112] reported the characterization of a small molecule pan‐KRAS inhibitor, BI‐2865, which did not discriminate among various KRAS mutants. This agent effectively inhibited KRAS activation and downstream pathways in vitro. Vivo studies demonstrated that its structural analogue, BI‐2493, significantly attenuated tumor growth in mice harboring KRAS G12C, G12D, G12V mutations, with no apparent toxicity observed. These findings warrant further clinical investigation in patients with KRAS‐driven malignancies [112]. Moreover, tri‐complex RAS inhibitors exhibiting broad‐spectrum activity against both mutant and wild‐type RAS variants have revolutionized the field of RAS drug discovery. For instance, RMC‐7977 has demonstrated potent activity against RAS‐addicted PDAC models [113]. Another tri‐complex RAS inhibitors, including RMC‐4998 and RMC‐6236, have also elicited significant tumor regression with favorable tolerance [114, 175]. Currently, RMC‐6236 is undergoing clinical evaluation in patients with KRAS‐mutant solid tumors, including advanced PDAC (NCT05379985).

In addition to the inherent technical challenges in identifying specific inhibitors, the development of acquired resistance represents a significant barrier to the efficacy of KRAS‐targeted therapies. Notably, acquired KRAS alteration and MET amplification confer resistance to adagrasib and sotorasib [176]. Furthermore, intratumoral heterogeneity and cellular variability are recognized as critical determinants of resistance to KRAS–G12C inhibitors. For example, Xue et al. [177] revealed that in NSCLC cells, treatment with a KRAS–G12C inhibitor induced a quiescent state characterized by diminished KRAS activity, while simultaneously promoting the synthesis of novel KRAS–G12C proteins, thereby fostering adaptive resistance. Additionally, activation of the PI3K/AKT/mTOR signaling pathway and the induction of EMT have been implicated in mediating resistance to sotorasib [178, 179, 180]. A comprehensive understanding of these mechanisms is essential for the development of innovative therapeutic approaches to overcome the resistance of KRAS inhibitors.

To enhance the efficacy of RAS inhibitors, comprehensive therapeutic approaches may provide benefit. For instance, as a downstream effector of RAS, active GTP‐bound RAS promotes RAF dimerization and phosphorylation, subsequently triggering RAF substrate phosphorylation [181]. VS‐6766, a pioneering RAF‐MEK inhibitor, demonstrated favorable efficacy and safety profiles in a phase I study across various cancers harboring RAF/RAS/MEK mutations [182]. Furthermore, the RHOA‐FAK axis serves as a pivotal downstream regulator in RAS pathway. Defactinib, a selective inhibitor of FAK, exhibited modest clinical efficacy in heavily pretreated NSCLC patients with mutated KRAS [183]. Notably, the combination therapy of VS‐6766 and defactinib demonstrated significant antitumor activity in patients with recurrent low‐grade serous ovarian cancer, particularly yielding an ORR of 73% among patients harboring KRAS mutation [184]. Given the manageable toxicity profile and promising clinical outcomes, further trials are warranted. Moreover, emphasis has been placed on the development of DNA vaccines, capable of inducing antitumor responses through the expression of tumor‐associated antigens (TAAs) and immunogens. Weng et al. [185] investigated the efficacy of a KRAS DNA vaccine in a lung cancer model driven by KRAS G12D, observing a significant reduction in tumor nodules alongside enhanced immune responses in vaccinated mice. Remarkably, mutant KRAS peptides have been evaluated as adjuvant treatment in patients with PDAC, revealing a significant improvement in 10‐year survival rates compared with nonvaccinated cohorts (20 vs. 0%) [186]. These findings underscore the promising potential of vaccines in the treatment of KRAS‐driven PDAC.

#### 3.2.3 Agents Targeting PARP Pathway

The DNA damage response (DDR) plays a critical role in preserving genomic stability. When DNA damage occurs, multiple pathways are activated to ensure accurate DNA repair or induce apoptosis, including homologous recombination (HR). Dysregulation or defects in these mechanisms can contribute to oncogenesis, making them promising therapeutic targets in cancer treatment [187]. Over the past decade, extensive research has established that mutations in BRCA1/2 genes result in HR deficiency (HRD), which in turn promotes uncontrolled cell proliferation and tumorigenesis [188]. Notably, PDAC ranks as the third most common malignancy associated with BRCA mutations, with approximately 5–10% of familial PDAC cases harboring these genetic alterations [189]. Moreover, BRCA mutations are strongly correlated with a significantly elevated risk of developing PDAC [190].

BRCA mutated cells rely on PARP as an alternative DNA repair mechanism. Farmer et al. [191] demonstrated that BRCA‐deficient cells are particularly susceptible to PARP inhibition (PARPi), which induces cell cycle arrest and triggers apoptotic cell death. This observation led to the conceptualization of synthetic lethality, wherein PARPi selectively targets cells with BRCA mutations. Olaparib, a pan‐inhibitor of PARP1, PARP2, and PARP3, has been extensively studied in this context. In the landmark POLO trial, olaparib was evaluated as maintenance therapy in PDAC patients with germline BRCA1/2 mutations who had not experienced disease progression following first‐line chemotherapy. The PFS was significantly prolonged in the olaparib group compared with the placebo group (7.4 vs. 3.8 months, *p* = 0.04) [192]. Emerging preclinical evidence further supports the therapeutic potential of olaparib in PDAC. Quiñonero et al. [193] reported that olaparib not only reduced the viability of PC cells but also significantly enhanced their sensitivity to GEM. Additionally, innovative strategies have been developed to optimize treatment efficacy, such as an EGFR‐targeting peptide nanoparticle designed for the codelivery of GEM and olaparib in BRCA2‐mutated PDAC. This delivery system demonstrated robust synergistic effects both in vitro and in vivo, underscoring its therapeutic potential [194]. Collectively, these findings highlight the promise of PARPi, either as monotherapy or in combination regimens, for the treatment of PDAC, particularly in patients with BRCA mutations.

Furthermore, next‐generation PARPis, such as niraparib, rucaparib, and talazoparib, have received approval for patients with BRCA‐mutant breast and ovarian cancer [195]. Significant efforts have also been toward evaluating their efficacy in PDAC. Maintenance rucaparib has been proposed as a safe and effective therapy for platinum‐sensitive advanced PDAC with BRCA1/2 mutation, resulting in a mPFS of 13.1 months and a mOS of 23.5 months, respectively [196]. Ongoing trials, such as NCT04550494, are investigating the efficacy of talazoparib in advanced cancer, including PC. In addition, venadaparib, the next‐generation PARP inhibitor, has demonstrated significant antitumor activity in preclinical PC models [115]. These promising results prompt the initiation of further phase Ib/IIa studies to evaluate the efficacy and safety of venadaparib.

One of the primary challenges associated with PARPi is the low mutation rate of BRCA in PDAC. Thus, it is imperative to expand the application of PARPi to patients who do not possess BRCA mutations. Furthermore, germline alterations in other DDR genes, such as ATM, PALB2, and CHEK2, significantly contribute to tumorigenesis [197]. Consequently, the exploration of these DDR genes as potential therapeutic targets warrants further investigation. In addition, acquired resistance to first‐generation PARPis remains a challenge. Previous studies have demonstrated that the ATR inhibitor ceralasertib could effectively restore sensitivity to olaparib in high‐grade serous ovarian cancer [198, 199]. This finding necessitates further validation in clinical practice. Moreover, the safety profile of PARPis requires improvement, as treatment can lead to hematological toxicity [200]. Accumulating evidence indicates that the selective trapping of PARP1, rather than PARP2, is sufficient to induce synthetic lethality in cancer cells with HRD [201]. And, PARP2 plays a crucial role in hematopoietic renewal. Therefore, its inhibition may result hematological AEs associated with first‐generation PARPis [202, 203]. In this context, PARP1‐selective inhibitors, such as saruparib (AZD5305), are currently developed. A preclinical study conducted by Herencia‐Ropero et al. [116] demonstrated its antitumor activity in murine models with HRD, thereby laying the groundwork for subsequent clinical trials. The ongoing phase I/II PETRA trial is evaluating the safety and efficacy of saruparib in patients with previously treated HRR‐deficient breast, ovarian, pancreatic, or prostate cancer (NCT04644068). The latest results indicated that among 31 breast cancer patients treated with 60 mg of saruparib, the ORR was 48.4%, with a median duration of response of 7.3 months and a mPFS of 9.1 months [204]. We eagerly anticipate the forthcoming responses in PDAC.

Furthermore, combining PARPi with other therapies is strongly recommended to enhance efficacy in PDAC. Previous studies suggested that maintenance niraparib plus ipilimumab was effective in advanced PDAC, while niraparib plus nivolumab had inferior outcomes [205]. These findings underscore the potential of noncytotoxic maintenance therapies in this context. Now, several ongoing trials are exploring the efficacy and safety of PARPi combined with immune checkpoint inhibitors (ICIs). Moreover, the combination of veliparib and FOLFOX demonstrated promising antitumor activity in advanced PDAC, particularly in patients with HR‐DDR mutations [206]. Results from another phase I trial supported the combination of veliparib and CRT as a promising strategy in locally advanced PC (LAPC) [207].

#### 3.2.4 Agents Targeting VEGF/VEGFR Pathway

VEGF/VEGFR serve as pivotal regulators governing vascular permeability, angiogenesis, and the development of pathological angiogenesis in tumor. VEGF induces the proliferation and survival of endothelial cells by binding to both VEGFR‐1 and VEGFR‐2, thereby fostering invasive tumor growth and distant metastasis [208].

Diverse strategies have emerged to target the VEGF/VEGFR pathway. Bevacizumab, a mAb against VEGF, has gained approval in multiple cancers [209]. However, its efficacy in PDAC is not promising. While a phase II trial demonstrated potential antitumor activity when combining bevacizumab with GEM in advanced PDAC [210], subsequent phase III trial failed to show positive results [211]. Similar outcomes were reported in other trials, as the addition of bevacizumab to GEM‐erlotinib did not significantly enhance OS in advanced PDAC [212]. Ramucirumab, a human IgG1 mAb targeting VEGFR‐2, has gained approval for treating advanced gastric cancer and gastro‐esophageal junction adenocarcinoma [213]. However, a phase II study investigating its combination with mFOLFIRINOX in advanced PDAC revealed no significant improvement of PFS or OS [214].

Regarding multitarget TKIs, sorafenib and regorafenib have been evaluated in PDAC. Sorafenib combined with docetaxel demonstrated synergistic efficacy in vitro and in vivo, suggesting a promising strategy for PDAC [117]. However, these findings lack further support from clinical trials. The phase II RESOUND study, enrolling patients with various refractory cancer types treated with regorafenib, reported a PDAC cohort with an 8‐week PFS rate of 25% and a mPFS of 1.7 months, failing to meet the primary endpoint. Hence, further exploration of regorafenib monotherapy in this context is not advised [215].

Sunitinib, primarily targeting VEGFR and platelet‐derived growth factor receptor (PDGFR), is approved for advanced renal cell carcinoma and gastrointestinal stromal tumor [216]. Preclinical study have indicated that sunitinib sensitized PC cells to radiation [118]. However, its combination with GEM did not demonstrate superiority over GEM monotherapy in advanced PDAC [217]. Similarly, the addition of axitinib, a highly selective inhibitor of VEGFR1/2/3, to GEM did not confer a survival benefit in advanced PDAC [218]. In sum, there remains significant to optimize the application of VEGF/VEGFR inhibitors for PDAC.

#### 3.2.5 Agents Targeting RAF/MEK/ERK Pathway

Targeting the RAF/MEK/ERK signaling pathway has emerged as a promising strategy for PDAC. Among RAF inhibitors, LY3009120, a pan‐RAF inhibitor, has exhibited significant antiproliferative effects in preclinical models of BRAF‐mutated and KRAS‐mutated CRC [219]. However, its clinical application in PDAC remains under investigation, with further trials needed to evaluate its safety and efficacy. Beyond LY3009120, multikinase inhibitors targeting oncogenic kinases, including RAF, have been extensively explored. For example, regorafenib, the second‐generation multikinase inhibitor, has demonstrated broad antitumor activity through the inhibition of various kinases, including RAF. Nevertheless, its efficacy in PDAC remains uncertain, necessitating additional research to elucidate its role in this challenging malignancy.

Another attractive therapeutic avenue involves targeting MEK, a key downstream kinase in the MAPK signaling pathway, which is frequently activated by oncogenic KRAS mutations. Trametinib, an oral MEK1/2 inhibitor, has shown promise in PC cells, particularly in those with KRAS–G12C mutation and low EGFR expression. These findings suggest that trametinib may be more effective in specific subtypes [119]. Other MEK inhibitors, such as CI‐1040 and PD0325901, have also been evaluated in phase I/II clinical trials. However, the results were modest, with limited clinical benefit [220, 221]. This hindered their further development and highlights the challenge of targeting the MAPK pathway.

To address these limitations, a promising therapeutic strategy has emerged: simultaneously targeting multiple nodes within the MAPK signaling pathway. This approach, which involves the combined inhibition of RAF, MEK, and ERK, aims to disrupt compensatory feedback mechanisms that frequently drive resistance to single‐agent therapies and diminish their overall efficacy. Ozkan‐Dagliyan et al. [26] conducted a study highlighting the synergistic efficacy of dual RAF and ERK inhibition in KRAS‐mutant PDAC. This combination effectively suppressed downstream ERK activation while concurrently mitigating the negative feedback loops that often compromise the efficacy of RAF inhibitors as monotherapy. Notably, at lower doses, this combination therapy exhibited pronounced synergistic effects, markedly enhancing antitumor effect compared with single‐agent inhibition of RAF, MEK, or ERK. These findings underscore the potential of simultaneously targeting multiple nodes within the MAPK pathway as a promising therapeutic strategy for KRAS‐mutant PDAC [26].

#### 3.2.6 Agents Targeting Insulin‐Like Growth Factor Pathway

Comprising insulin alongside two associated ligands, insulin‐like growth factor receptor (IGF) ligand 1 and 2 (IGF‐1 and IGF‐2), the IGF axis significantly contributes to tumorigenesis [222]. The IGF‐1 receptor (IGF‐1R), a RTK, is activated by IGF‐1 and IGF‐2. After activation, IGF‐1R activates signaling cascades, including PI3K/AKT/mTOR and RAS/RAF/MEK/ERK [223]. Existing evidence underscores the intimate association between the IGF pathway and PDAC [224]. For instance, Karna et al. [225] found that in the serum and tissues of PDAC patients, the IGF‐1 and IGF‐binding proteins were increased. Meanwhile, vitro experiments demonstrated that the administration of IGF‐1 promoted the proliferation of PC cells [226]. Notably, this effect can be abrogated by inhibiting IGF‐1R, rendering it a promising therapeutic target. Three strategies have been proposed for targeting the IGF pathway: receptor blockade via mAbs, kinase inhibitors, and ligand sequestration.

NVP‐AEW541 is a representative of small molecular kinase inhibitor of IGF‐1R, which have much higher affinity to IGF‐1R. Its antitumor activity has been identified in vitro and in vivo. Study performed by Manara et al. [227] found that NVP‐AEW541 could against the growth of Ewing's sarcoma cells, as well as angiogenesis. In the case of PC, Moser et al. [120] demonstrated that with administration of NVP‐AEW541, the activation of IGF‐IR and ERK/AKT in PC cells was diminished. In vivo, NVP‐AEW541 significantly reduced the growth of orthotopic pancreatic tumor. Hence, targeting IGF‐1R with NVP‐AEW541 is a potential treatment for PDAC.

As for mAbs, ganitumab (AMG 479) stood out. First, it can inhibit the binding of IGF‐1 and IGF‐2 to IGF‐1R, thereby effectively suppressing cell proliferation. Second, ganitumab impedes the activation of IGF‐IR homodimers and IGF‐IR hybrid receptors, as opposed to INSR homodimers. Furthermore, ganitumab has been demonstrated to downregulate IGF‐IR expression [121, 228]. Previous study revealed that it robustly inhibits IGF‐1‐mediated AKT phosphorylation in PC xenograft models, highlighting its therapeutic potential in modulating the AKT signaling pathway [121]. Encouraged by these preclinical findings, clinical trials were initiated to evaluate its efficacy and safety. A phase Ib study established the acceptable safety profile of ganitumab when administered in combination with GEM for the treatment of untreated advanced PDAC [229]. However, subsequent phase III trial yielded disappointing results. When the combination of ganitumab and GEM was administrated as first‐line treatment for advanced PDAC, the outcomes were not improved [230]. Meanwhile, cixutumumab (IMC‐A12), another mAb targeting IGF‐1R, exhibited antitumor effects in preclinical studies when combined with various chemotherapeutic drugs [231]. Nevertheless, a phase Ib/II study evaluating cixutumumab in combination with erlotinib and GEM for advanced PDAC did not demonstrate improved outcomes [232].

In summary, strategies targeting the IGF pathway remain an unmet clinical need, with significant challenges hindering their efficacy. These include dose‐limiting toxicities and suboptimal antitumor activity, which limited the clinical utility of anti‐IGF therapies. For example, the insulin receptor (IR) and IGF‐1R exhibit a high degree of structural and functional homology. This redundancy enables compensatory activation, where inhibition of one receptor may trigger the other to assume its signaling role, thereby serving as a resistance mechanism [233]. Consequently, dual targeting of IR and IGF‐1R may elicit a synergistic antitumor effect, offering a strategy to circumvent adaptive resistance to single‐agent IGF‐1R inhibitors.

#### 3.2.7 Agents Targeting NOTCH Pathway

The NOTCH pathway plays pivotal roles in various aspects of tumor biology. Additionally, it modulates adaptive immune responses by influencing tumor‐associated macrophages (TAMs), myeloid suppressor cells (MDSCs), DCs, and other immune cells [234]. Upregulation of the NOTCH pathway has been observed in precancerous lesions, highlighting its potential as a therapeutic target [235].

Demcizumab (OMP‐2 1M18), a humanized mAb targeting DLL4, disrupts the interaction between DLL4 and the NOTCH receptor. DLL4 is often overexpressed in tumor cells, potentially activating the NOTCH pathway. Demcizumab inhibits DLL4 through three main mechanisms: suppressing tumor stem cell, promoting nonfunctional angiogenesis, and regulating immune response [236]. Phase I trials investigating demcizumab in patients with solid tumors indicated that it was well tolerated, warranting further evaluation in phase II studies [237, 238, 239]. A phase I trial (NCT01189929) was initiated to assess demcizumab in combination with GEM with or without abraxane as first‐line treatment for advanced PDAC. However, no results have been published yet.

Tarextumab (OMP‐59R5), a dual antagonist of NOTCH‐2 and NOTCH‐3, was assessed in a PDX model of PC by Yen et al. [122]. The findings revealed that the tumor significantly regressed when tarextumab was combined with GEM–NabP. Subsequent gene set enrichment analysis unveiled the upregulation of EMT gene sets in GEM‐treated tumors, which was reversed by tarextumab combined therapy [122]. Consequently, clinical trials were initiated to investigate the efficacy of tarextumab in combination with GEM–NabP for advanced PDAC [240, 241]. However, the phase II trial results were disappointing, with the triplet regimen (tarextumab + GEM–NabP) exhibiting worse mPFS compared with its competitor (GEM–NabP) [241]. Discrepancies between preclinical findings in PDX model and clinical outcomes in patient populations may contribute to these discordance.

γ‐Secretase is a multisubunit transmembrane protein complex, comprising both catalytic and accessory subunits, all of which contain transmembrane domains essential for its structural integrity and functional activity [242]. Through γ‐secretase‐mediated activation, NOTCH ligands engage receptors, culminating in the formation of the activated ICN, which translocates to the nucleus to regulate pivotal genes regulating cell proliferation and differentiation [123]. γ‐Secretase inhibitors (GSIs) primarily impede the cleavage of ICN domains, thereby preserving NOTCH in an intact, latent state [243]. Preclinical investigations have underscored the antitumor efficacy of GSIs in murine models of PDAC, either as monotherapy or in combination with GEM [123, 244]. MK‐0752, a highly potent and selective GSI, underwent evaluation in a phase I trial involving patients with advanced solid tumors. Clinical benefit was observed, and toxicity was dose dependent [245]. Concurrently, another study evaluated the combination of MK‐0752 and GEM in PDAC patients. Among the 19 eligible patients for response assessment, a DCR of 73.7% was reached. Given that the efficacy of the combined regimen was comparable with that of GEM monotherapy, further investigation of this combination is not justified [243]. RO4929097, another GSI, yielded a mOS of 4.1 months and a mPFS of 1.5 months in a phase II trial involving patients with advanced PDAC [246]. Although the development of this agent was terminated, the trial laid the groundwork for GSIs‐based combination therapies in solid tumors, including PDAC.

#### 3.2.8 Agents Targeting TGF‐β

Evidence supports the involvement of the TGF‐β pathway in the development and progression of PC. Galunisertib, a small molecule inhibitor of TGF‐β receptor I (TGF‐βRI) kinase, selectively downregulates the phosphorylation of SMAD2 and negates the activation of classical pathway. A study performed by Yingling et al. [247] demonstrated its antitumor activity in preclinical models by inhibiting TGF‐βRI and SMAD phosphorylation, while also reversing TGF‐β‐mediated immune suppression. These findings were substantiated by further investigation. In the 4T1 mouse model, galunisertib exhibited dose‐dependent antitumor activity with a concomitant increase of T cells. Its combination with a PD‐L1 inhibitor demonstrated synergistic efficacy in colon carcinoma models [248]. The favorable outcomes facilitated the clinical exploration of galunisertib. In a phase II trial, the combination of galunisertib and GEM in patients with advanced PDAC demonstrated improved OS, compared with GEM monotherapy (8.9 vs. 7.1 months) [249]. Conversely, in another phase II trial, its combination with durvalumab (an anti‐PD‐L1 antibody) exhibited limited efficacy in heavily pretreated PDAC, with a DCR of 25% [250].

As a TGF‐βI receptor/ALK‐5 kinase inhibitor, vactosertib has gained attention. In PC, its synergy with GEM elicited antitumor activity both in vitro and in vivo. The mechanism may contribute to the inhibition of ECM and the TGF‐β/SMAD2 pathway [124]. Presently, a phase II trial is underway to assess the safety of vactosertib combined with liposomal irinotecan, 5‐FU, and leucovorin in patients with advanced PDAC (NCT04258072). Fresolimumab (GC1008) emerges as a pan‐TGF‐β mAb. A phase I trial enrolling patients with malignant melanoma and renal cancer, demonstrated the antitumor activity of fresolimumab with manageable safety. Notably, one patient achieved PR, and six patients maintained SD, with a mPFS of 24 weeks [251], suggesting the potential of fresolimumab as a disease‐specific therapy.

#### 3.2.9 Agents Targeting Cyclin‐Dependent Kinase 4/6

Cyclin‐dependent kinase 4/6 (CDK4/6) regulates cell cycle by interacting with Cyclin D to drive the G1/ S phase transition, thereby promoting cell proliferation. Dysregulation of CDK4/6 is a critical driver in cancer [252]. CDK4/6 inhibitors have emerged as promising targeted therapies, with three inhibitors (palbociclib, ribociclib, and abemaciclib) approved for clinical practice [253].

Preclinical evidence suggested that CDK4/6 inhibition holds potential for treating PC. For example, palbociclib induces G1‐phase arrest in PC cells, reduces cell viability while promoting an epithelial phenotype and suppressing mesenchymal markers [125]. Given the resistance of PC cells‐particularly those with KRAS mutations‐to monotherapies, combination strategies are being investigated. In vitro, the combination of the HDAC inhibitor panobinostat and abemaciclib induces synergistic apoptosis, surpassing the effects of either drug alone [128]. Moreover, in a PDX model of PC, palbociclib combined with NabP showed better efficacy than GEM–NabP. Based on this, a phase Ib trial was performed to assess the efficacy and safety of palbociclib plus NabP in advanced PDAC. The results demonstrated antitumor activity, with a 50% 12‐month survival rate and manageable AEs, but the predefined efficacy threshold was not met [126]. Ribociclib, another CDK4/6 inhibitor, is approved for postmenopausal women with hormone receptor‐positive, HER2‐negative breast cancer [254].

CDK4/6 has also been implicated in tumor progression through interactions with pathways such as PI3K/AKT [255], supporting the rationale for combination therapies with other targeted agents. A phase I trial combining CDK4/6 inhibitors with mTOR inhibitor in chemotherapy‐resistant PDAC showed good tolerance but limited efficacy as third‐line treatment [256]. Additionally, combining palbociclib with the ERK inhibitor ulixertinib was found to synergistically inhibit the growth of PC cell lines and organoids by counteracting the compensatory upregulation of ERK, PI3K, and antiapoptotic signals induced by CDK4/6 inhibition [127]. A phase I clinical trial (NCT03454035) is now evaluating the combination of ERK and CDK4/6 inhibitors in advanced PDAC, with results pending.

Importantly, CDK4/6 inhibitors may modulate the TME by affecting immune cell functions, such as T cells, potentially enhancing the immune response against tumors [257, 258]. Thus, combining CDK4/6 inhibitors with immunotherapy represents a promising strategy. Although investigations into CDK4/6 inhibitors in PDAC are still in early stages, emerging preclinical evidence indicates that their combination with chemotherapy or immunotherapy could offer novel opportunities.

Here, we summarized agents targeting diverse signaling pathways, with available clinical data (Table 3). Despite advances in targeted therapy, the translation of basic research findings into clinical applications for PDAC continues to face significant challenges.

**TABLE 3:** Agents targeting different signaling pathways with available clinical data in pancreatic ductal adenocarcinoma.

Phase	Identifier	Setting	Treatment	Outcomes	References
III	—	Advanced	Erlotinib + GEM vs. GEM	mOS: 6.24 vs. 5.91 months; *p* = 0.038	[130]
I	—	LAPC	RT + GEM + gefitinib	mOS: 7.5 months; mPFS: 3.7 months	[132]
II	—	Advanced	Docetaxel + gefitinib	TTP: 1.8 months; OS: 4.5 months	[133]
II	—	Advanced	Docetaxel + gefitinib	TTP: 2.1 months; OS: 2.9 months	[134]
II	NCT01728818	Advanced	Afatinib + GEM vs. GEM	mOS: 7.3 vs. 7.4 months; *p* = 0.80 mPFS: 3.9 vs. 3.9 months; *p* = 0.43	[136]
II	NCT00536614	Advanced	Cetuximab + GP vs. GP	mOS: 7.5 vs. 7.8 months; *p* = 0.739 mPFS: 3.4 vs. 4.2 months; *p* = 0.847	[141]
II	—	Advanced	GEMOXCET	TTP: 118 days; OS: 213 days	[142]
II	—	Advanced	Cetuximab + docetaxel + irinotecan vs. docetaxel + irinotecan	mOS: 5.4 vs. 6.5 months; mPFS: 4.5 vs. 3.9 months	[143]
I/II	NCT00923299	Advanced	Cetuximab + trastuzumab	mOS: 4.6 months;; mPFS: 1.8 months	[148]
II	—	Advanced	Nimotuzumab	mOS: 18.1 weeks; mPFS: 6.7 weeks	[150]
IIb	NCT00561990	Advanced	Nimotuzumab + GEM vs. GEM	mOS: 8.6 vs. 6.0 months; *p* = 0.0341 mPFS: 5.1 vs. 3.4 months; *p* = 0.0163	[151]
III	NCT02395016	Advanced	Nimotuzumab + GEM vs. GEM	mOS: 10.9 vs. 8.5 months mPFS: 4.2 vs. 3.6 months	[152]
I/II	NCT03600883	Advanced	Sotorasib	mOS: 6.9 months; mPFS: 4.0 months	[159]
II	NCT03785249	Advanced	Adagrasib	mOS: 8.0 months; mPFS: 5.6 months	[169]
I/II	NCT05009329 NCT05002270	Advanced	Glecirasib	ORR: 46.4%; DCR: 96.4%	[171]
III	NCT02184195	Maintenance	Olaparib vs. placebo	mOS: 18.9 vs. 18.1 months; *p* = 0.68 mPFS: 7.4 vs. 3.8 months; *p* = 0.004	[192]
II	NCT03140670	Maintenance	Rucaparib	mOS: 23.5 months; mPFS: 13.1 months	[196]
Ib/II	NCT03404960	Maintenance	Group A: Niraparib + nivolumab Group B: Niraparib + ipilimuma	Group A mOS: 13.2 months; mPFS: 1.9 months Group B mOS: 17.3 months; mPFS: 8.1 months	[205]
I/II	NCT01489865	Advanced	Veliparib + FOLFOX	ORR: 26%	[206]
I	NCT01908478	Advanced	Veliparib + GEM + RT	mOS: 15 months	[207]
II	—	Advanced	Bevacizumab + GEM	mOS: 8.8 months; mPFS: 5.4 months	[210]
III	—	Advanced	Bevacizumab + GEM vs. GEM	mOS: 5.8 vs. 5.9 months; *p* = 0.95 mPFS: 3.8 vs. 2.9 months; *p* = 0.07	[211]
III	—	Advanced	Bevacizumab + GEM + erlotinib vs. GEM + erlotinib	mOS: 7.1 vs. 6.0 months; *p* = 0.2087 mPFS: 4.6 vs. 3.6 months; *p* = 0.0002	[212]
II	NCT02581215	Advanced	Ramucirumab + mFOLFIRINOX vs. mFOLFIRINOX	mOS: 10.3 vs. 9.7 months; *p* = 0.094 mPFS: 5.6 vs. 6.7 months; *p* = 0.322	[214]
II	NCT02307500	Advanced	Regorafenib	mPFS: 1.7 months	[215]
II	—	Advanced	Sunitinib + GEM vs. GEM	mOS: 30.4 vs. 36.7 weeks; *p* = 0.78 mPFS: 11.6 vs. 13.3 weeks; *p* = 0.78	[217]
III	NCT00471146	Advanced	Axitinib + GEM vs. GEM	mOS: 8.5 vs. 8.3 months; *p* = 0.5436	[218]
III	NCT01231347	Advanced	Ganitumab (12 mg/kg) + GEM vs. ganitumab (20 mg/kg) + GEM vs. placebo + GEM	mOS: 7.0 vs. 7.1 vs. 7.2 months mPFS: 3.6 vs. 3.7 vs. 3.7 months	[230]
Ib//II	NCT00617708	Advanced	Cixutumumab + erlotinib + GEM vs. erlotinib + GEM	mOS: 7.0 vs. 6.7 months; *p* = 0.64 mPFS: 3.6 vs. 3.6 months; *p* = 0.97	[232]
II	NCT01647828	Advanced	Tarextumab + NabP + GEM vs. NabP + GEM	mOS: 6.4 vs. 7.9 months; *p* = 0.0985 mPFS: 3.7 vs. 5.5 months; *p* = 0.04	[241]
I	NCT01098344	Advanced	MK‐0752 + GEM	DCR: 73.7%	[243]
II	—	Advanced	RO4929097	mOS: 4.1 months; mPFS: 1.5 months	[246]
Ib/II	—	Advanced	Galunisertib + GEM vs. GEM	mOS: 8.9 vs. 7.1 months	[249]
Ib	NCT02734160	Advanced	Galunisertib + durvalumab	DCR: 25%; mOS: 5.72 months; mPFS: 1.87 months	[250]

*Abbreviations*: GEM, gemcitabine; OS, overall survival; LAPC, locally advanced PC; PFS, progression‐free survival; RT, radiotherapy; TTP, time to progression; GP, gemcitabine + cisplatin; GEMOXCET, cetuximab plus gemcitabine/oxaliplatin; ORR, objective response rate; DCR, disease control rate; NabP, nab‐Paclitaxel.

### 3.3 Immunotherapy

**FIGURE 2:**
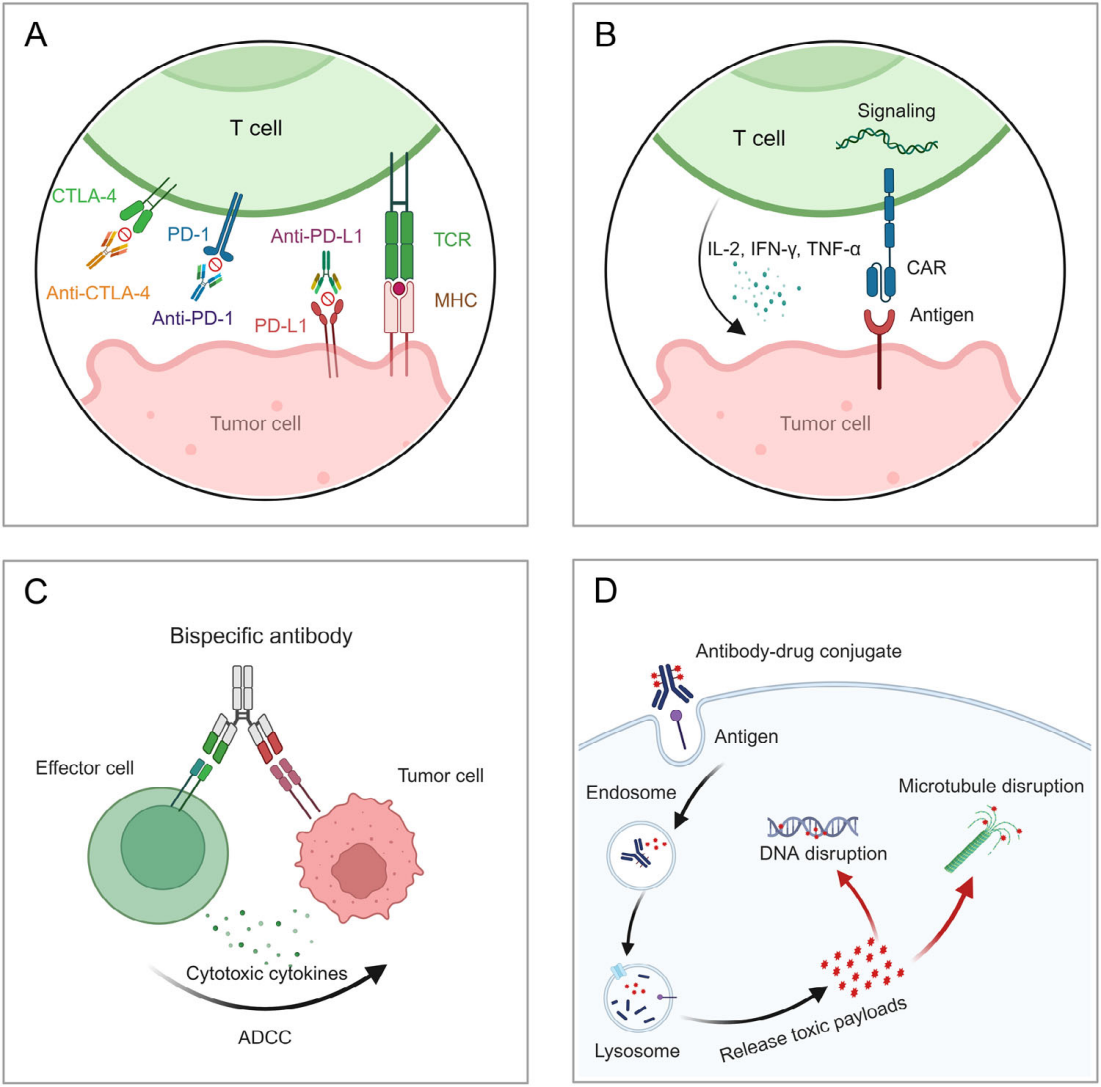
Mechanisms of various therapies. (A) Anti‐PD‐1/PD‐L1 antibodies inhibit the PD‐1/PD‐L1 axis and promote a positive immune response to eliminate the tumor. The antibody against CTLA‐4 functions to sustain T cell activation by releasing inhibitory signals that otherwise impede T cell activation. (B) CARs comprise an extracellular scFv or VHH serving as the targeting moiety, a transmembrane spacer, and intracellular signaling or activation domains. These CAR constructs are introduced into T cells using various transduction systems, effectively directing T cells to recognize and bind to surface‐expressed tumor antigens. (C) Bispecific antibodies possess dual antigen‐binding arms, with one arm binding to tumor cell target antigens and the other binding to marker antigens on effector cells. This dual interaction activates effector cells, facilitating their targeting and subsequent elimination of tumor cells. (D) Antibody–drug conjugates specifically target tumor antigens, facilitating their endocytosis into tumor cells. Once internalized, ADCs are transported to lysosomes, where they release their toxic payloads. The released payloads can induce apoptosis by disrupting DNA or microtubule and may also exert a bystander effect, leading to the elimination of adjacent tumor cells. *Abbreviations*: PD‐1/PD‐L1, programmed cell death protein 1 and its ligands; CTLA‐4, cytotoxic T‐lymphocyte antigen‐4; TCR, T cell receptor; MHC, major histocompatibility complex; CAR, chimeric antigen receptor; ADCC, antibody dependent cell mediated cytotoxicity (Created with BioRender.com, with permission).

The past decade has witnessed notable achievements in immunotherapy (Table 4). Cytotoxic T‐lymphocyte antigen‐4 (CTLA‐4), a subset of costimulatory molecules present on T cell surfaces, interacts with B7 molecules on antigen‐presenting cells, thereby impeding the activation of T cell. This inhibitory signal could be countered by anti‐CTLA‐4 antibodies. Moreover, programmed cell death protein 1 (PD‐1) expresses on T cells, while its ligand expresses on tumor cells. This interaction leads to T cell depletion and suppression of T cell‐mediated antitumor activity. Second‐generation ICIs targeting the PD‐1/PD‐L1 axis disrupt this signaling pathway, maintaining the antitumor efficacy of T cells (Figure 2A).

**TABLE 4:** Key trials of immunotherapy in pancreatic ductal adenocarcinoma.

Trial	Phase	Identifier	Setting	Treatment	Outcomes	References
—	II	—	Advanced	Ipilimumab	No response	[259]
—	I	NCT00729664	Advanced	BMS‐936559 (PD‐L1 antibody)	No objective response	[260]
KEYNOTE‐158	II	NCT02628067	Advanced (MSI‐H/dMMR)	Pembrolizumab	ORR: 18.2%	[261]
—	II	ChiCTR2000032955	Neoadjuvant	Tislelizumab + CRT	ORR: 60%; R0 resection rate: 90%	[262]
CISPD‐4	II	NCT03983057	Neoadjuvant	mFOLFIRINOX + PD‐1 antibody vs. mFOLFIRINOX	PR rate: 36.3 vs. 13.0%	[263]
—	—	—	Neoadjuvant	mFOLFIRINOX + nivolumab	mPFS: 34.8 months; mOS: 35.1 months	[264]
—	I	NCT02309177	Advanced	Nivolumab + GEM–NabP	mPFS: 5.5 months; mOS: 9.9 months	[265]
—	Ib	NCT01473940	Advanced	Ipilimumab + GEM	mPFS: 2.78 months; mOS: 6.90 months	[266]
—	I	NCT02546531	Advanced	Defactinib + pembrolizumab + GEM	DCR: 80%	[267]
—	Ib/II	ChiCTR2000032293	Advanced	Toripalimab + GEM–NabP	mPFS: 5.6 months; mOS: 8.9 months	[268]
—	II	NCT02704156	Locally recurrent	SBRT + GEM vs. SBRT + pembrolizumab + trametinib	mOS: 12.8 vs. 14.9 months; *p* = 0.0021	[269]
CheckPAC	II	NCT02866383	Advanced	SBRT + nivolumab vs. SBRT + nivolumab + ipilimumab	CBR: 17.1 vs. 37.2%	[270]
COMBAT	IIa	NCT02826486	Advanced	Cohort 1: BL‐8040 + pembrolizumab Cohort 2: BL‐8040 + pembrolizumab + chemotherapy	DCR: Cohort 1: 34.5% Cohort 2: 77%	[271]
—	I/Ib	NCT03874897 NCT04581473	Advanced	CT041 (Claudin18.2‐specific CAR‐T)	mPFS: 3.3 months; mOS: 10.0 months	[272]
—	Ib	NCT01413022	Borderline resectable or LAPC	PF‐04136309 (CCR2 inhibitor) + FOLFIRINOX	ORR: 49% DCR: 97%	[273]
—	Ib	NCT02732938	Advanced	PF‐04136309 + GEM–NabP	ORR: 23.8%	[274]

*Abbreviations*: MSI‐H, high microsatellite instability; dMMR, DNA mismatch repair; ORR, objective response rate; CRT, chemoradiotherapy; PR, partial response; GEM–NabP, gemcitabine + nab‐Paclitaxel; DCR, disease control rate; SBRT, stereotactic body radiotherapy; CBR, clinical benefit rate; CAR‐T, chimeric antigen receptor T cell.

#### 3.3.1 Immune Checkpoint Inhibitors

The persistent discrepancy in survival benefits across different cancers and PDAC is particularly notable. ICI monotherapy has shown limited antitumor efficacy. For instance, in a phase II trial, advanced PDAC patients showed no response to single‐agent ipilimumab (anti‐CTLA‐4) [259]. Similarly, in a phase I trial, the administration of an anti‐PD‐L1 antibody yielded no objective response in patients with PC [260]. In KEYNOTE‐158 trial, noncolorectal patients with microsatellite‐unstable (MSI‐H) or mismatch repair‐deficient (dMMR) were administrated with pembrolizumab, including advanced PDAC. Benefit was observed in these patient population, as ORR achieved 18.2% [261]. Despite this, it is important to highlight that MSI‐H/dMMR represent a very small subset of PDAC, which may restrict the application for the general population [275].

Several factors have been implicated as contributors to this phenomenon, including high tumor heterogeneity and immunosuppressive TME [276]. Considering the prominent role of chemotherapy and radiotherapy in PDAC, their combination with ICIs emerges as a promising therapy. In the neoadjuvant setting, a phase II study assessed the combination of PD‐1 blockade (tislelizumab) and chemoradiotherapy as preoperative therapy for patients with locally advanced or borderline resectable PDAC. The ORR was 60%, with an R0 resection rate of 90%. The 12‐month PFS and OS rates were 64 and 72%, respectively. ctDNA analysis revealed that patients with a >50% reduction in maximal somatic variant allelic frequency (maxVAF) had longer survival, higher response rates, and increased surgical resection rates. These findings suggested that PD‐1 blockade combined with chemoradiotherapy has promising antitumor activity, and biomarkers could help identify patients who may benefit most [262]. Another trial (NCT03983057) compared neoadjuvant mFOLFIRINOX with mFOLFIRINOX plus PD‐1 antibody for borderline resectable and locally advanced PDAC. Preliminary results indicated radiological PR in 13.3% of patients in the chemotherapy group and 26.9% in the PD‐1 group. For borderline resectable PDAC, the PR rates were 13.0 and 36.3%, respectively. The resection rates were similar between two groups (47.4 vs. 51.7%), with R0 resections achieved in 70.3 and 86.6%, respectively. In LAPC, the PD‐1 group had a higher resection rate (48.0 vs. 37.1%). Given the feasibility and tolerability of the combination regimen, the study is still ongoing [263]. Furthermore, results from a pilot trial evaluating neoadjuvant mFOLFIRINOX plus nivolumab in borderline resectable PDAC were presented. The mPFS was 34.8 months, and mOS was 35.1 months. Among patients undergoing resection, the 18‐month OS rate was 90%, with two cases of pathologic complete response (CR). The addition of nivolumab did not increase the incidence of grade 3 AEs. RNA sequencing suggested that nivolumab may enhance antitumor efficacy by promoting cytolytic T‐cell function and reducing immunosuppressive adenosine signaling. A subsequent phase II trial is ongoing [264].

As for advanced setting, a phase I trial evaluated the safety and efficacy of nivolumab in combination with GEM–NabP. However, the efficacy was modest, failing to warrant further investigation [265]. Similarly, the addition of ipilimumab to GEM did not confer superiority over GEM monotherapy in advanced PDAC [266]. In contrast, the combination of defactinib, pembrolizumab, and GEM demonstrated favorable safety profiles and efficacy in patients with refractory PDAC, achieving a DCR of 80% [267]. In addition, a phase Ib/II study has made significant strides in the field of immunotherapy for advanced PDAC, which evaluated the safety and efficacy of toripalimab combined with GEM–NabP as first‐line treatment. In 72 patients, the mOS was 8.9 months, with a 12‐month OS rate of 31.9%. The mPFS was 5.6 months, with an ORR of 33.3% and a DCR of 90.3%. Additionally, cyclic multiplex tissue staining assay and artificial intelligence‐driven spatial analysis were employed to identify patients most likely to benefit from immunotherapy. The findings revealed that the enrichment of DC‐helper T cell‐cytotoxic T lymphocyte (DC‐Th‐CTL) immune domains and their spatial interactions may serve as key predictive biomarkers [268]. These findings are warranted to validate in further studies. In addition, evidence suggests that radiotherapy may augment the effectiveness of immunotherapy. For instance, compared with stereotactic body radiotherapy (SBRT) plus GEM, the combination of SBRT with pembrolizumab and trametinib significantly improved outcomes in patients with locally recurrent PDAC, achieving a mOS of 14.9 months compared with 12.8 months with SBRT plus GEM group (*p* = 0.0021) [269]. Consistent results were observed in other studies, demonstrating clinical benefit from the combination of nivolumab and ipilimumab with SBRT in advanced PDAC [270].

Another strategy to enhance the sensitivity of PDAC to ICIs is through their synergistic combination with inhibitors targeting the CXC chemokine receptor (CXCR) axis. Evidence suggests that the TME of PDAC is regulated by the CXCR4‐CXCL12 axis, and inhibiting this pathway may promote the infiltration of T cells to TME, thereby augmenting tumor sensitivity to ICIs [277, 278]. Based on this, Bockorny et al. [271] conducted a phase II trial to evaluate the efficacy of the CXCR4 antagonist, BL‐8040, in combination with pembrolizumab and chemotherapy for advanced PDAC. The results found that patients receiving this triple‐combination therapy achieved an ORR of 32% and a DCR of 77% [271]. LY2510924 is another selective peptide antagonist of CXCR4. A phase I study assessed its combination with durvalumab in patients with advanced refractory solid tumors, including PDAC. Best overall response of SD was observed in three advanced PDAC. However, due to the small sample size, its evidence‐based foundation requires further validation [272].

Currently, ICIs are utilized in a limited subset of advanced PDAC, with several ongoing studies investigating the combination strategies involving ICIs, the outcomes of which are eagerly anticipated (Table 5). It is worth noting that the safety profile associated with these aggressive therapies has garnered considerable attention. It is crucial to enhance efficacy while ensuring safety. Several strategies could be employed. For instance, the utilization of biomarkers can aid in identifying individuals who are likely to benefit from specific immunotherapies, thereby minimizing ineffective treatments and potential AEs [279]. Additionally, real‐time monitoring of AEs represents an effective approach to bolstering safety [260]. Lastly, during the design of clinical trials, it is imperative to emphasize safety monitoring to ensure that potential AEs are identified and addressed early in the study, thereby safeguarding patients’ well‐being and treatment quality.

**TABLE 5:** Ongoing clinical trials evaluating immunotherapies in pancreatic ductal adenocarcinoma.

NCT	Phase	Setting	Treatment	Primary outcomes
NCT06435260	II	Neoadjuvant	Camrelizumab, GEM–NabP, hypofractionated radiotherapy	R0 resection rate
NCT02451982	II	Neoadjuvant	CY/GVAX vs. CY/GVAX + nivolumab vs. CY/GVAX + nivolumab + urelumab vs. BMS‐986253 (anti‐IL8 antibody) + nivolumab	IL17A expression Intratumoral CD8+CD137+cells Intratumoral granzyme B+PD‐1+CD137+ cells Pathologic response
NCT05604560	I	Neoadjuvant	Tislelizumab + SX‐682 (CXCR1/2 inhibitor)	Pathologic response rate
NCT05721846	I	Neoadjuvant	Nivolumab, ipilimumab, TGFβ‐15 peptide vaccine, SBRT	AEs
NCT03727880	II	Neoadjuvant/adjuvant	Pembrolizumab + defactinib vs. pembrolizumab	Pathologic complete response rate
NCT03983057	III	Neoadjuvant	FOLFIRINOX and Anti‐PD‐1 antibody vs. FOLFIRINOX	PFS
NCT04117087	I	Adjuvant	KRAS vaccine peptide + nivolumab + ipilimumab	AEs
NCT05726864	I/II	Adjuvant (mutated KRAS/NRAS)	ELI‐002 7P	Safety
NCT05419479	I/II	Maintenance	Domvanalimab + zimberelimab + APX005 M vs. FOLFIRINOX	DLTs, PFS
NCT04548752	II	Maintenance (germline mutation in BRCA 1/2)	Olaparib + pembrolizumab vs. olaparib	PFS
NCT04753879	II	Maintenance	GAX‐CI + maintenance of oembrolizumab and olaparib	PFS after 6 months
NCT05955157	II/III	Maintenance	DC‐CIK + S‐1 vs. S‐1	AEs
NCT05088889	I	Maintenance	Ipilimumab + nivolumab	ORR
NCT03806309	II	Maintenance	OSE2101 (vaccine) + FOLFIRI vs. FOLFIRI	OS
NCT04887805	II	Maintenance	Pembrolizumab + lenvatinib	PFS
NCT04543071	II	Advanced	Motixafortide, cemiplimab, GEM–NabP	Overall response rate
NCT06359275	II	Advanced	Toripalimab + GEM–NabP + PULSAR	PFS
				
NCT05927142	I/II	Advanced	Durvalumab + rintatolimod (TLR‐3 agonist)	Safety and Clinical benefit rate
NCT05632328	II	Advanced	Cohort 1: AGEN1423 (anti‐CD73‐TGFβ‐Trap bifunctional antibody), + botensilimab (anti‐CTLA‐4 antibody) Cohort 2: AGEN1423 + botensilimab + GEM–NabP	ORR
NCT06051851	II	Advanced	Penpulimab + anlotinib + GEM–NabP vs. GEM–NabP	PFS
NCT05451043	II	Advanced	Durvalumab + tremelimumab + propranolol + GEM–NabP	Efficacy
NCT01174121	II	Advanced	Experimental: 1/CD8+ enriched TIL Experimental: 2/unselected TIL Experimental: 3/unselected TIL + pembrolizumab prior to cells Experimental: 4/unselected TIL + pembrolizumab at POD	Response rate
NCT06411691	I	Advanced	mKRAS vaccine + botensilimab + balstilimab	ORR
NCT05102721	I/II	Advanced	Avelumab + pepinemab	AEs
NCT05630183	II	Advanced	Botensilimab + GEM–NabP vs. GEM–NabP	PFS
NCT06333314	II	Advanced (dMMR/MSI)	Dostarlimab vs. chemotherapy	PFS
NCT05411094	I	Advanced	Olaparib + durvalumab + RT	MTD/RP2D
NCT05580445	Ib/II	Advanced	FAK inhibitor + toripalimab + GEM	RP2D
NCT05681390	II	Advanced	Tislelizumab + anlotinib + chemotherapy	PFS
NCT05218889	I/II	Advanced	Surufatinib + camrelizumab + NabP + S‐1 vs. GEM–NabP	DLTs, RP2D, ORR
NCT05620732	/	Advanced	Claudin18.2 CAR‐T	AEs
NCT05539430	I	Advanced	Claudin18.2 CAR‐T	RDE, RP2D
NCT05605197	I	Advanced	U87 CAR‐T	AEs
NCT06196658	I	Advanced	EX02 CAR‐T	AEs
NCT05438667	I	Advanced	KRAS mutant antigen specific TCR‐T	OS, PFS
NCT04146298	I/ II	Advanced	mutant KRAS G12V‐specific TCR‐T	AEs
NCT06054984	I	Advanced	TCR‐T Cells	AEs
NCT03104439	II	MSI‐H PDAC	Nivolumab + ipilimumab + RT	DCR

*Data sources*: Clinical registration website.

*Abbreviations*: GEM–NabP, gemcitabine + nab‐Paclitaxel; CY, cyclophosphamide; SBRT, stereotactic body radiotherapy; AEs, adverse events; PFS, progression‐free survival; DLTs, dose‐limiting toxicities; GAX‐CI, gemcitabine, nab‐paclitaxel, capecitabine, cisplatin, and irinotecan; DC+CIK: dendritic cell + cytokine‐induced killer cell; ORR, objective response rate; OS, overall survival; PULSAR, personalized ultrafractionated stereotactic adaptive radiation therapy; TILs, tumor‐infiltrating lymphocytes; RT, radiotherapy; MTD, maximum tolerated dose; RP2D, recommended phase 2 dose; FAK, focal adhesive kinase; CAR‐T, chimeric antigen receptor T cell; dMMR, deficient mismatch repair; MSI‐H, microsatellite instability high; RDE, recommended dose for expansion; TCR‐T, T cell receptor T cell; PDAC, pancreatic ductal adenocarcinoma; DCR, disease control rate.

#### 3.3.2 Chimeric Antigen Receptor T Cell Therapy

Chimeric antigen receptor T cell (CAR‐T) therapy involves the fusion of an anticancer mAb with intracellular T cell receptor (TCR) signaling domains (Figure 2B) [280]. While CAR‐T therapy has gained approval for hematological malignancies, its efficacy in solid tumors has been constrained. However, recent investigations have shown promising efficacy of Claudin 18.2 (CLDN18.2) CAR‐T therapy in PDAC. CLDN18.2, a tight junction protein predominantly expressed in epithelial cells, exhibits abnormal expression patterns during malignancy progression. Evidence suggests that 59.2% of PDAC patients harbor CLDN18.2‐positive cells [281]. For patients with advanced PDAC receiving CT041, a genetically engineered autologous T cells expressing CLDN18.2‐targeted CAR, lung metastasis achieved CR [282]. The latest pooled analysis indicates that among PDAC patients treated with CT041, the overall response rate is 16.7%, and DCR is 70.8%. The mPFS is 3.3 months, while the mOS is 10.0 months, collectively underscoring the efficacy of CT041 [283]. Relative mechanism has been explored. Takasawa et al. [284] identified a positive feedback loop between CLDN18 and ERK1/2 in bile duct cancer, while another study elucidated how hyperoxia disrupted alveolar epithelial cell tight junctions via upregulation of the SPAK–MAPK pathway, leading to the reduced expression of CLDN18 [285]. In PC cells, CLDN18 transcriptional upregulation occurs through specific PKC pathways and is subject to modification via DNA methylation [286]. Despite the promising results achieved by CLDN18.2 CAR‐T therapy in clinical practice, further exploration is needed to enhance the efficacy and address the tumor heterogeneity.

#### 3.3.3 Outlooks

Barriers to effective immunotherapies in PDAC encompass various factors, with the accumulation of immunosuppressive cells and desmoplastic stroma posing a critical obstacle to T cell infiltration. Therefore, the imperative to refine the immunosuppressive TME is evident, prompting the consideration of several strategies. For instance, efforts to ‘heat’ cold tumors include disrupting the CXCR4/CXCL12 axis, inhibiting FAK, and targeting the upstream JAK–STAT pathway [287]. Previous findings indicated that hyperactivated FAK activity in PDAC cells served as a crucial regulator of the fibrotic and immunosuppressive TME. Administration of a FAK inhibitor significantly suppressed tumor progression in vivo, leading to reduced tumor fibrosis and diminished infiltration of immunosuppressive cells. Furthermore, FAK inhibition enhanced sensitivity to T cell immunotherapy and PD‐1 antagonists, thereby positioning it as a promising candidate for combination with immunotherapy [288].

Moreover, as mentioned above, the TME in PDAC represents a significant barrier to the successful application of immunotherapy. Various cellular components, including Tregs, MDSCs, and TAMs, foster immune tolerance [289, 290]. The intricate interplay of cytokines and metabolites within the TME contributes to the proliferation of immunosuppressive cells, which inhibits the function of effective immune cells. Notably, elevated expression of colony‐stimulating factor 1 (CSF1) and its receptor (CSF1R) is associated with limited efficacy of ICIs [290]. Therefore, blockade of CSF1R may enhance the efficacy of immunotherapy. In a preclinical study, Zhu et al. [291] observed that the combination of CSF1R inhibitor with PD‐1 inhibitor and chemotherapy significantly inhibited tumor growth, compared with PD‐1 inhibitor plus chemotherapy. A phase I trial (NCT02777710) evaluating the safety and efficacy of the CSF1R inhibitor pexidartinib in combination with durvalumab in patients with advanced CRC and PDAC reported PR in one CRC patient with high microsatellite instability, while 26% tumor reduction lasting 3.6 months in one PDAC patient. Although the study revealed limited antitumor activity, the researchers noted that pexidartinib impaired DC differentiation via inhibiting FLT3 signaling, suggesting potential avenues for optimizing this combination therapy in the future [292].

Additionally, TAMs are primarily recruited to the TME through the chemokine ligand 2 (CCL2) and chemokine receptor 2 (CCR2) signaling axis, which in turn suppresses CD8+ T cell activation. MDSCs are also recruited to the TME via the CCL2‐CCR2 pathway, further exacerbating immune suppression [290]. Consequently, targeting the CCL2‐CCR2 axis may help restore immune function. A phase I trial assessing the CCR2 inhibitor PF‐04136309 in combination with FOLFIRINOX demonstrated favorable safety and efficacy, achieving an ORR of 49% and a DCR of 97% in a cohort of borderline resectable and locally advanced PDAC [273]. However, subsequent phase Ib/IIa study combining PF‐04136309 with GEM–NabP was terminated due to insufficient efficacy and synergistic pulmonary toxicity [274].

Regarding CAR‐T therapy, tumor‐specific antigens (TSAs) and TAAs hold significant importance. Several antigens, such as CEACAM7, MUC16, and trophoblast cell surface antigen 2 (Trop2), have been identified in PC. CEACAM7, a member of the CEA protein family, is known to be overexpressed in pancreatic tissue. Raj et al. [293] developed and evaluated a CAR targeting CEACAM7 for PDAC, demonstrating its therapeutic efficacy in vitro and in vivo. Moreover, Trop2 is expressed in various solid tumors. A T2‐CAR targeting Trop2 was developed, showing potent anticancer efficacy against Trop2‐positive cells. Furthermore, the incorporation of the CD27 intracellular domain enhanced the killing effect of T2‐CAR‐T cells by upregulating IL‐7Rα expression and downregulating PD‐1 expression [294]. Notably, Trop2‐targeted CAR‐T cells efficiently infiltrated tumor tissues and significantly suppressed the growth of pancreatic xenograft tumor [295].

Besides, there are other immunotherapies with great potential. Supported by robust research, the replacement of T lymphocytes with natural killer (NK) cells for CAR expression is one of possible solutions. As early as 2020, Li et al. [296] reported a case of advanced PDAC with liver metastasis who received Robo1‐specific CAR‐NK therapy. This treatment was well tolerated and resulted in a OS of 8 months [296]. The aberrant overexpression of prostate stem cell antigen (PSCA) is prevalent in PC, with an incidence ranging from 60 to 80% [297]. Consequently, Teng et al. [298] undertook the engineering of PSCA CAR‐NK cells and conducted assessment of its efficacy both in vitro and in vivo. The findings substantiated its therapeutic efficacy in PDAC models, exhibiting a favorable safety profile. These results furnished a robust rationale for further clinical exploration [298]. Conversely, major histocompatibility complex (MHC) class I‐related proteins A (MICA) and B (MICB) manifest heightened expression across various solid tumors. FT536, derived from a clonal master induced pluripotent stem cell (iPSC) line, encompasses four functional elements, notably including a novel CAR designed to selectively target the α3 domain of MICA and MICB [299]. Presently, a phase I trial evaluating the safety and efficacy of FT536 in diverse advanced solid tumors (e.g., NSCLC, gastric cancer, and PC) is underway (NCT05395052). We are looking for the results.

Comparatively, TCR‐engineered T cell (TCR‐T) therapy emerges as an appealing alternative in the context of solid tumors. Leidner et al. [300] documented a case wherein TCR‐T targeting mutant KRAS G12D yielded a PR, sustained over a period of 6 months. Concurrently, multiple trials investigating the efficacy and safety of TCR‐T in PDAC are ongoing, hoping to establish a novel therapeutic cornerstone in the treatment of PDAC (Table 5).

### 3.4 Radiotherapy

Over decades, radiotherapy has been extensively investigated in PDAC. Its efficacy as a neoadjuvant treatment was evaluated in the PREOPANC‐1 and A021501 trials, which yielded inconsistent results, potentially due to different radiation modalities. The role of radiotherapy in the adjuvant setting also remains controversial. Long‐term follow‐up data from the EORTC 40891 trial revealed no significant difference in OS between PDAC patients receiving postoperative CRT and those under observation. However, the statistical validity of these findings is limited by the incomplete treatment completion in 20% of the CRT group [301]. Similarly, the ESPAC‐1 trial failed to substantiate the benefits of radiotherapy in postoperative adjuvant therapy [65]. In contrast, a large‐scale retrospective study demonstrated a notable improvement in OS (21.2 vs. 14.4 months, *p* < 0.001) and 5‐year survival rates (20.1 vs. 15.4%) among patients treated with CRT, compared with those who were not [302]. Consistently, Hsu et al. [303] reported that adjuvant CRT with 5‐FU was associated with significantly prolonged OS compared with surgery alone (mOS, 21.1 vs. 15.5 months, *p* < 0.001), with enhanced survival outcomes after adjusting for variables such as age, resection margins, lymph node status, and T‐stage.

Additionally, the RTOG 9704 trial evaluated the efficacy of integrating GEM into fluorouracil‐based CRT on postoperative 5‐year OS in PDAC. Although the addition of GEM did not significantly improve OS, multivariable analysis revealed a trend toward enhanced OS in patients with tumors localized to the head of the pancreas [304]. These findings provided the foundational rationale for the subsequent RTOG 0848 trial. The most recent results from RTOG 0848 demonstrated that CRT did not improve OS but did enhance DFS. In lymph node‐negative patients, CRT improved both OS and DFS. Compared with chemotherapy alone, CRT did not increase the incidence of grade 4–5 AEs [305].

For advanced disease, the FFCD/SFRO study performed by Chauffert et al. [306] to compare CRT with chemotherapy alone in LAPC. However, the combined approach, involving an intensive induction schedule of CRT followed by maintenance GEM, failed to improve outcomes and led to increased toxicities [306]. Conversely, another study reported improved OS with the addition of radiotherapy to GEM in LAPC patients, with manageable toxicities [307]. Furthermore, the long‐term results of the SCALOP trial confirmed the superiority of CRT in LAPC [308]. Recent advancements in radiotherapy techniques have further optimized therapeutic efficacy. Notably, intensity‐modulated radiotherapy has been associated with a more favorable safety profile compared with conventional 3D conformal radiotherapy in the management of PDAC [309, 310]. Additionally, SBRT has demonstrated significant potential in overcoming the inherent radio‐resistance of PDAC. A systematic review underscored the benefits of SBRT, including reduced treatment duration, improved OS, and enhanced 1‐year locoregional control rates, establishing it as a viable therapeutic option for inoperable PDAC [311]. Moreover, the combination of SBRT with FOLFIRINOX has been shown to increase the likelihood of achieving radical surgical resection in LAPC patients [312]. Concurrently, stereotactic magnetic resonance‐guided adaptive radiotherapy has emerged as a promising modality for unresectable PDAC, with preliminary data indicating encouraging clinical outcomes [313, 314].

Carbon ion radiotherapy (CIRT) has gradually attracted attention in various cancers. Unlike proton irradiation, the efficacy of carbon ions is relatively independent from the oxygenation of the irradiated tissue and cell cycle phase, which is supposed to induce higher relative biological effectiveness (RBE) with more accurate dose distribution [315, 316]. Moreover, preclinical study indicated that CIRT may enhance the infiltration of immune cells, and upregulate immune checkpoint molecules (e.g., PD‐1, LAG‐3, and TIM‐3). As CIRT increased the expression of PD‐L1, its combination with PD‐L1 inhibitor showed synergistic effect in vivo, providing groundwork for this combined strategy [317]. Currently, there are several clinical trials showing promising results in advanced PDAC treated with CIRT. In a single‐institutional study, LAPC patients treated with definitive CIRT achieved a median survival of 25.1 months, with 82 and 23% 2‐year OS and PFS rates, respectively [318]. The results were in line with another multi‐institutional study, as patients with LAPC treated with CIRT achieved a mOS of 21.5 months. The 1‐ and 2‐year distant metastasis‐free survival (DMFS) rates were 41 and 28%, respectively. These outcomes were associated with a favorable safety profile, characterized by limited toxicities [319]. Furthermore, very preliminary findings have provided evidence supporting the feasibility of combining chemotherapy with CIRT in the neoadjuvant setting of resectable and borderline resectable PDAC [320]. Future investigations into novel combination strategies incorporating CIRT hold significant promise for improving therapeutic outcomes in PDAC.

Nevertheless, CIRT is associated with several limitations. First, the substantial costs associated with facility construction and maintenance impose a significant economic burden, thereby restricting its widespread clinical application. Second, the determination of baseline doses and the accurate calculation of RBE remain technically challenging. Due to the heightened RBE of CIRT, meticulous attention is required to minimize the deposition of carbon ions in nontarget tissues, thereby reducing unintended toxicity. Additionally, the selection of lymph node regions for irradiation remains an area requiring further investigation, particularly in the context of CIRT combined with chemotherapy, as well as in the precise delineation of target volumes.

### 3.5 Emerging Therapies

In recent years, a new wave of innovative therapies has emerged, offering hope for improving patient outcomes. These emerging therapies, including antibody–drug conjugates (ADCs), bispecific antibodies (BsAbs), cancer vaccines, and oncolytic viruses (OVs), target key aspects of pancreatic tumor biology, immune evasion mechanisms, and TME, with the goal of enhancing treatment efficacy.

#### 3.5.1 Bispecific Antibodies

BsAbs have revolutionized therapeutic strategies for advanced malignancies, leveraging their unique mechanisms to demonstrate a broad spectrum of clinical applications (Figure 2C). First, BsAbs can effectively redirect immune effector cells, such as T cells and NK cells, toward tumor cells. A notable example is bispecific T‐cell engagers (BiTEs), which simultaneously bind to TSAs and the CD3 subunit on T cells. This dual binding facilitates T‐cell activation and subsequent tumor cell lysis [321]. Second, BsAbs can target two distinct epitopes or antigens, enabling the simultaneous blockade of multiple signaling pathways. For instance, BsAbs targeting both EGFR and HER2 (anti‐EGFR × anti‐HER2) or EGFR and c‐MET (anti‐EGFR × anti‐c‐MET) exemplify this approach, highlighting their potential to disrupt oncogenic signaling networks [322, 323].

Considerable efforts have been dedicated to the development of BsAbs. Catumaxomab, a BsAb directed against EpCAM and CD3, demonstrates a unique ability to bind three different cell types: tumor cells, T cells, and accessory cells [324]. Umebayashi et al. [325] identified that cancer stem‐like cells (CSLCs) contributed to chemoresistance in PDAC. Pre‐treatment with catumaxomab, followed by the addition of interleukin‐2/OKT3, effectively activated autologous T cells, leading to the elimination of CSLCs in vitro. This approach presents a promising therapeutic strategy for chemotherapy‐resistant pancreatic CSLCs [325]. Zenocutuzumab, a BsAb targeting HER2 and HER3, has shown encouraging results in NRG1 fusion‐positive PDAC. In a clinical trial involving 12 PDAC patients treated with zenocutuzumab, the ORR reached 42%, with 50% of patients achieving SD [326]. These findings were further corroborated by a study conducted by Schram et al. [327], which confirmed clinical responses in two patients with ATP1B1‐NRG1‐positive PDAC. Cadonilimab, a BsAb targeting PD‐1 and CTLA‐4, has demonstrated substantial efficacy across multiple cancer types. It has recently received approval for the treatment of recurrent or metastatic cervical cancer in patients who have progressed on prior platinum‐based chemotherapy [328]. Notably, several ongoing clinical trials (NCT06532617, NCT06405490, NCT06153368) are investigating the combination of cadonilimab with other therapies for PDAC.

Despite high expectations, BsAbs have yet to achieve significant breakthroughs in PDAC, contrasting their remarkable success in leukemia and lymphoma. The identification of TSAs and TAAs, such as EpCAM, HER family members, CEA, and PSMA, represents a critical step in this endeavor. Notably, prior research has demonstrated that the addition of ipilimumab significantly enhances cytokine secretion and the specific cytotoxic activity of T cells armed with BsAbs [329]. These findings suggest that combining BsAbs with immunotherapeutic agents may represent a promising strategy for PDAC.

#### 3.5.2 Antibody–Drug Conjugates

ADCs are a class of therapeutic agents comprising a mAb covalently linked to a potent cytotoxic payload via a chemical linker. Compared with conventional chemotherapeutic agents, ADCs are distinguished by their highly specific target and enhanced antitumor efficacy, enabling the precise and efficient elimination of cancer cells (Figure 2D). Furthermore, their relatively small molecular size contributes to superior tissue penetration within the TME, enhancing their efficacy [330].

Presently, several ADCs have received approval for cancer treatment. Trastuzumab deruxtecan (DS‐8201) comprises an anti‐HER2 antibody, a cleavable tetrapeptide‐based linker, and a cytotoxic topoisomerase I inhibitor. In the phase II DESTINY‐Breast01 trial, DS‐8201 demonstrated durable antitumor activity in heavily pretreated patients with HER2‐positive metastatic breast cancer, with a 60.9% response rate and a mPFS of 16.4 months [331]. Subsequent phase III trial confirmed these findings [332]. However, in the DESTINY‐PanTumor02 study enrolling patients with HER2‐expressing solid tumors, no objective response was observed in the first 15 advanced PDAC patients with HER2 immunohistochemistry (IHC) 3+ expression. With regard to this, this cohort was closed [333]. The underlying mechanism remains undisclosed, highlighting a crucial gap in knowledge.

In addition to the aforementioned ADCs, several others with distinct compositions and mechanisms have emerged as pivotal players in cancer therapeutics, including trastuzumab emtansine (T‐DM1), enfortumab vedotin, and sacituzumab govitecan [330]. Decades of research have focused on optimizing the key components of ADCs, leading to the development of numerous promising candidates. Among these, OBI‐999 is a novel ADC composed of a mAb specific to globo H (GH), conjugated to the potent cytotoxic agent monomethyl auristatin E (MMAE) via a site‐specific ThioBridge and a cleavable linker. GH is highly overexpressed in several malignancies, including PC. Preclinical study performed by Yang et al. [334] demonstrated that OBI‐999 significantly inhibited tumor growth in a pancreatic xenograft model, highlighting its potential efficacy. Clinically, Tsimberidou et al. [335] conducted a phase I trial of OBI‐999 monotherapy in patients with advanced cancer. Among the 15 enrolled patients, five achieved SD with manageable AEs. However, in the subsequent phase II cohort‐expansion study involving patients with pancreatic, colorectal, and other GH‐expressing cancers, OBI‐999 failed to demonstrate the anticipated efficacy. Consequently, the trial was terminated (NCT04084366).

ICAM1, a transmembrane glycoprotein, is significantly upregulated in PC [336]. Emerging evidence indicates that KRAS mutations drive the overexpression of ICAM1 in pancreatic acinar cells, positioning ICAM1 as a promising therapeutic target [337]. ICAM1‐DM1 (Mertansine), a highly selective ADC, has demonstrated efficacy by effectively infiltrating the TME through ICAM1 targeting while exhibiting minimal toxicity in a PC murine model. To further advance precision medicine, Huang et al. [338] developed a noninvasive MRI‐based molecular imaging approach capable of identifying ICAM1 expression in PC patients. Additionally, ICAM1 holds potential for broader therapeutic applications, including CAR‐T cell therapy and BsAbs targeting ICAM1. This is particularly relevant as KRAS mutation‐induced ICAM1 overexpression recruits macrophages, accelerating the formation of precancerous lesions [337].

DUMC5754A, an ADC comprising a humanized anti‐MUC16 mAb conjugated to MMAE, exhibits potent and selective antitumor activity in PC murine model with MUC16‐expression [339]. Despite this promising preclinical efficacy, there exists limited clinical evidence supporting its effectiveness in patients with advanced PDAC [340]. CLDN18.2 is a key therapeutic target in digestive cancers, and the anti‐CLDN18.2 ADC, IBI343, has demonstrated encouraging efficacy in patients with advanced PDAC and biliary tract cancers (NCT05458219). Recent data showed that advanced PDAC patients with elevated CLDN18.2 expression may benefit from IBI343 treatment, which is associated with a manageable safety profile. PRs were observed in five PDAC patients. Among 10 PDAC patients, the ORR was 40%. These findings underscore the potential of IBI343 as a novel approach for this patient population [341].

Despite their promise, several critical challenges hinder widespread clinical application of ADCs. These include the identification of optimal target antigens, the incorporation of highly effective cytotoxic payloads, the development of advanced linker technologies, and the complex synthesis and manufacturing processes of mAbs. Addressing these challenges has driven significant efforts. For instance, the selection of appropriate antigens remains the fundamental in ADC design. Currently, several ADCs targeting antigens like HER2, Trop2, EGFR, CD19, and BCMA have received approval [342]. Among emerging targets, leucine‐rich repeat‐containing protein 15 (LRRC15), which mediates cell–cell and cell–matrix interactions, has shown promise as a therapeutic target for cancer‐associated fibroblasts and the TME [343]. ABBV‐085, an anti‐LRRC15 ADC, demonstrated antitumor activity in preclinical studies across multiple solid tumors, including PC [344, 345]. In a phase I clinical trial, ABBV‐085 exhibited preliminary antitumor efficacy in sarcoma patients, with manageable AEs, warranting further investigation in PDAC [346]. Additionally, preclinical studies have provided a strong rationale for combining ADCs with immunotherapies, spurring the design of clinical trials exploring their synergistic effects in solid tumors [347, 348]. Encouraging results have been observed in diverse malignancies, including breast, lung, urothelial, and ovarian cancers [349, 350, 351, 352]. These findings underscore the potential to optimize combination strategies and develop innovative therapeutic treatments for PDAC.

#### 3.5.3 Oncolytic Viruses

OVs, which leverage the unique ability of viruses to selectively infect and lyse cancer cells while sparing normal tissues, have garnered substantial attention as a promising strategy for PDAC. Recent advancements in OVs have focused on overcoming the formidable challenges posed by PDAC, such as its dense fibrotic stroma, immunosuppressive TME, and intrinsic resistance to conventional therapies [353].

Several OVs have advanced to clinical evaluation, demonstrating their therapeutic potential in PDAC. VCN‐01, an oncolytic adenovirus specifically engineered to replicate in RB1‐deficient cancer cells and express hyaluronidase, exhibited significant antitumor activity in preclinical PDAC models, particularly in combination with chemotherapy. However, a phase I clinical trial revealed that intratumoral administration of VCN‐01 combined with chemotherapy, while well tolerated, yielded limited efficacy, with all patients experiencing disease progression due to the emergence of new or enlarging metastatic lesions [354]. In another study, LOAd703, a genetically modified adenovirus coexpressing TMZ‐CD40L and 4‐1BBL, was evaluated in a phase I/II trial for advanced PDAC. The combination of LOAd703 with chemotherapy demonstrated a favorable safety profile, with common AEs including fever, fatigue, and transient liver dysfunction. Notably, 94% of patients exhibited an increase in CD8+ T cell infiltration, and among 18 evaluable patients, 44% achieved an objective response, highlighting the potential of this combined strategy [355]. Further exploration of LOAd703 in combination with atezolizumab is currently underway in an ongoing clinical trial (NCT02705196).

HF10, a spontaneously attenuated oncolytic herpes simplex virus‐1 (HSV‐1), has demonstrated potent antitumor activity in clinical trials for LAPC. In a phase I study, the combination of HF10 with erlotinib and GEM, administered under endoscopic ultrasound guidance, exhibited a favorable safety profile with a mPFS of 6.3 months and a mOS of 15.5 months. These outcomes suggest the therapeutic potential of HF10 in PDAC, warranting further investigation in larger patient cohorts [356]. Similarly, pelareorep, a proprietary reovirus isolate, has shown promising clinical benefits in PDAC. Combined with GEM, pelareorep achieved a mOS of 10.2 months, with 1‐ and 2‐year survival rates of 45 and 24%, respectively, while maintaining a manageable toxicity profile. Further analysis revealed upregulated expression of PD‐L1, highlighting a compelling rationale for combining pelareorep with ICIs [357]. In a follow‐up study, the addition of pembrolizumab to pelareorep and chemotherapy demonstrated promising efficacy, suggesting that this triple combination could provide enhanced benefits for advanced PDAC [358].

Despite these advancements, significant barriers persist in the application of OVs for PDAC, particularly the dense stromal environment and the immunosuppressive TME, which impede effective viral delivery and promote immune‐mediated viral clearance. To address these challenges, innovative strategies are under development, including the engineering of OVs to express immune‐stimulatory molecules. One promising example is Ad5‐yCD/mutTKSR39rep‐hIL12, an oncolytic adenovirus engineered to express human interleukin‐12 (hIL‐12) and suicide genes. The suicide genes are enzymatically converted into their respective prodrugs, leading to irreversible inhibition of DNA synthesis and robust antitumor activity. Furthermore, the expression of hIL‐12 enhances the antitumor immune response. In a phase I dose‐escalation study, Ad5‐yCD/mutTKSR39rep‐hIL12 demonstrated favorable tolerance in patients with locally recurrent prostate cancer [359]. A subsequent phase I clinical trial (NCT03281382) was conducted to evaluate its efficacy in advanced PDAC, although results have not yet been published. Furthermore, the potential synergistic effects between OVs and immunotherapy represent a pivotal area of investigation. Combining OVs with ICIs, such as anti‐PD‐1 or anti‐CTLA‐4 antibodies, may amplify immune‐mediated tumor rejection, a particularly critical approach in the immunosuppressive TME of PDAC. Although direct intratumoral injection of OVs offers more targeted delivery compared with systemic intravenous administration, technical challenges persist due to the anatomical complexity of the pancreas and its dense stromal barriers. Future research efforts should focus on optimizing viral targeting strategies, enhancing immune responses, and ensuring safety profiles. These advancements have the potential to establish a therapeutic paradigm for PDAC.

#### 3.5.4 Cancer Vaccines

Cancer vaccines offer a promising strategy to enhance antitumor immunity by stimulating the host immune system to recognize and target tumor cells. They can also modulate the immunosuppressive TME, thereby improving T cell‐mediated antitumor responses. Currently, prophylactic vaccines for cervical and liver cancer are wildly used [360].

In recent years, multiple vaccine platforms have been explored in both preclinical and clinical settings, with varying degrees of success. For instance, long‐term follow‐up data from clinical trials of KRAS peptide vaccines showed that patients who received the vaccine postsurgery had a better 10‐year survival rate compared with those who did not [186]. However, the efficacy of cancer vaccine monotherapy in highly malignant cancers such as PDAC remains limited. As a result, researchers are exploring combination therapies. For example, GM‐CSF gene‐modified tumor vaccine (GVAX), a type of irradiated allogeneic tumor vaccine secreting GM‐CSF, was evaluated in resected PDAC patients. After surgery, patients received GVAX followed by 5‐FU‐based CRT and achieved a mDFS of 17.3 months and a mOS of 24.8 months. The regimen was well tolerated, warranting further investigation [361]. Algenpantucel‐L, an engineered allogeneic whole‐cell vaccine designed to activate complement and antibody‐dependent cell cytotoxicity, was also tested in a trial alongside CRT in resected PDAC. The 12‐month DFS rate was 62%, and the OS rate was 86% [362]. However, subsequent phase III trial did not meet its primary endpoints, failing to demonstrate improved survival outcomes when added to postsurgical CRT [363].

Furthermore, cancer vaccines hold potential when combined with other immunotherapies. For example, Wu et al. [364] conducted a trial exploring GVAX combined with ipilimumab as maintenance therapy for advanced PDAC. Patients, having received first‐line FOLFIRINOX chemotherapy, were randomly assigned to either GVAX with ipilimumab or continuation of FOLFIRINOX after 8–12 cycles, with ongoing response or SD. Although GVAX and ipilimumab promoted T cell differentiation into effector memory phenotypes and increased M1 macrophage infiltration, the combination did not improve OS compared with continued chemotherapy (9.38 vs, 14.7 months, *p* = 0.019) [364]. Similarly, a study by Zheng et al. [365] evaluated the combination of GVAX, pembrolizumab, and SBRT in LAPC. The primary endpoint was DMFS. The results indicated a mDMFS of 9.7 months, with 44% of patients subsequently undergoing surgical resection. Common treatment‐related AEs included injection site reactions, while grade 3 immune‐related AEs included dermatitis, colitis, and pneumonia [365]. Analysis of paired pre‐ and posttreatment specimens revealed that the combined strategy promotes a TME that favors antitumor immune responses, including increased CD8 T cells, T‐helper 1 (TH1), and TH17 cells. However, it also led to an increase in immunosuppressive M2‐like TAMs. These findings have provided valuable insights into the design of future studies aimed at targeting M2‐like TAMs in PDAC [366]. Additionally, the team investigated the perioperative efficacy of a GVAX‐based immunotherapy in resectable PDAC. Participants were randomized into three treatment arms: arm A receiving GVAX with low‐dose cyclophosphamide alone, arm B receiving GVAX plus nivolumab, and arm C receiving GVAX in combination with both nivolumab and the anti‐CD137 agonist antibody urelumab. The results demonstrated mPFS of 13.90, 14.98, and 33.51 months and mOS of 23.59, 27.01, and 35.55 months for arms A, B, and C, respectively. Although the triple combination of GVAX, nivolumab, and urelumab exhibited the most favorable survival outcomes, the differences did not reach statistical significance, likely due to the limited sample size [367]. Further phase III clinical trials are warranted to confirm the efficacy of this strategy.

The efficacy of cancer vaccines critically depends on the precise identification of high‐quality tumor antigens, which are indispensable for eliciting robust immune responses. In PDAC, KRAS mutations remain the most prominent therapeutic target. ELI‐002 2P, a novel lymph node‐targeting vaccine, has been specifically engineered to recognize KRAS G12D and G12R mutations. This vaccine is designed for patients with PDAC or CRC at high risk of relapse who exhibit molecular residual disease (MRD) following local therapies, with the primary objective of mitigating recurrence. The phase I AMPLIFY‐201 trial enrolled 20 PDAC patients and 5 CRC patients, all characterized by high relapse risk and MRD after surgical or chemotherapy [368]. Preliminary results demonstrated a median relapse‐free survival (mRFS) of 15.3 months and a mOS of 28.9 months in the PDAC subgroup. Notably, ELI‐002 2P exhibited a favorable safety profile, with no grade 3–4 AEs or cytokine release syndrome reported [369]. Building on the encouraging outcomes from AMPLIFY‐201, a phase II trial is currently in progress (NCT05726864).

Recent advances in precision medicine have driven the development of personalized cancer vaccines, leading to significant breakthroughs. For instance, Rojas et al. [370] synthesized an individualized neoantigen vaccine (autogene cevumeran) based on uridine mRNA lipid nanoparticles derived from surgically resected PDAC samples. This vaccine targets multiple unique neoantigens. After surgical resection, patients were administered a combination of atezolizumab, autogene cevumeran, and mFOLFIRINOX. T‐cell responses to the vaccine were evaluated using IFN‐γ ELISpot, revealing that 50% of patients exhibited significant T‐cell reactivity. Notably, long‐lasting specific CD8+ T‐cell responses were observed 2 years postsurgery. During follow‐up, vaccine responders demonstrated significantly prolonged mRFS compared with nonresponders (not reached vs. 13.4 months, *p* = 0.003). Regarding safety, all patients receiving autogene cevumeran experienced grade 1–2 AEs, with one case of grade 3 AE reported. Collectively, the combination of atezolizumab, autogene cevumeran, and mFOLFIRINOX elicited robust T‐cell activation, potentially preventing recurrence [370]. In the same year, a separate study evaluated an mRNA vaccine targeting the KRAS G12V mutation in solid tumors, including a case of LAPC. Due to the poor health condition, neither chemotherapy nor surgery was feasible. Remarkably, after three cycles of mRNA vaccination combined with pembrolizumab, significant regression of the pancreatic head lesion was observed, achieving a PR alongside a marked improvement in quality of life [371]. These studies underscore the therapeutic potential of neoantigen vaccines, offering promising strategies for both the management of advanced disease and the prevention of postsurgical recurrence.

Despite these advancements, significant challenges persist in the application of cancer vaccines. The major limitation lies in the immunosuppressive TME, where T‐cell exhaustion may impair the vaccine's capacity to elicit robust antitumor immune response. Furthermore, intratumoral heterogeneity and the dynamic acquisition of tumor mutations often facilitate immune evasion, rendering previously effective vaccines obsolete. To address these issues, combined therapies, such as integrating cancer vaccines with cytokine‐based adjuvants (e.g., IL‐2 or GM‐CSF), could potentially rejuvenate T‐cell activity and enhance immunogenicity. Additionally, advancements in delivery systems, including nanoparticles and liposomes, hold promise for improving antigen stability, cellular uptake, and bioavailability, thereby potentially augmenting vaccine efficacy in advanced disease.

## 4 Future Directions and Conclusions

In this review, we comprehensively discussed the latest advancements in PDAC, a malignancy characterized by high mortality rate. The lack of early diagnostic biomarkers and effective therapeutic strategies has significantly contributed to the persistently poor prognosis associated with this disease.

Although chemotherapy remains a cornerstone of PDAC treatment, recent insights into the biological mechanisms underlying tumorigenesis have catalyzed the development of targeted therapies and immunotherapeutic approaches, offering new avenues. Notably, emerging research on PARP inhibitors, ICIs, BsAbs, ADCs, and combination therapies has demonstrated potential in improving survival by targeting tumor‐specific mutations and the TME. Concurrently, the advent of precision medicine has underscored the importance of tailoring treatment strategies to individual molecular profiles. For patients with neurotrophin receptor kinase (NTRK) gene fusions, selective inhibitors such as larotrectinib and entrectinib is strongly recommended [372, 373]. Similarly, for those with RET fusion‐positive tumors, selpercatinib, a potent RET kinase inhibitor, represents a viable therapeutic option [374]. Additionally, the identification of genes associated with pyroptosis, ferroptosis, and cuproptosis has elucidated their roles in PDAC pathogenesis. Beyond their prognostic significance, these genes hold promise as predictive biomarkers for immunotherapy response in PDAC [375, 376, 377].

Emerging evidence increasingly highlights a critical role of the gut microbiome in modulating the progression of PDAC. Specific microbial populations within the pancreatic TME exhibit dynamic interactions with the gut microbiome, collectively shaping tumor biology and therapeutic outcomes. Notably, the composition and diversity of the gut microbiome have been shown to significantly influence treatment responses [378]. For instance, Sivan et al. [379]. demonstrated that distinct commensal microbial communities profoundly affect antitumor immunity in melanoma‐bearing mice. Utilizing 16S ribosomal RNA sequencing, they identified bifidobacterium as a key microbial species capable of enhancing antitumor immunity. When combined with anti‐PD‐L1 therapy, oral administration of bifidobacterium promoted DCs maturation and cytokine production, leading to increased recruitment of CD8+ T cells into the TME and subsequent suppression of tumor growth [379]. Similarly, Akkermansia muciniphila has been shown to augment the efficacy of ICIs in epithelial tumors [380]. These findings suggest that targeted modulation of the gut microbiota, potentially through dietary interventions, could serve as a promising strategy to enhance immunotherapy. In PDAC specifically, the microbiota‐derived tryptophan metabolite indole‐3‐acetic acid (3‐IAA) has been identified as enriched in patients responsive to chemotherapy. Preclinical study revealed that in murine models of PDAC, oral supplementation of 3‐IAA enhances the efficacy of chemotherapy through mechanisms involving neutrophil‐derived myeloperoxidase [381]. This underscores the potential of microbiome‐targeted nutritional interventions as adjuncts to conventional chemotherapy. Despite the lack of large‐scale clinical trials, the intricate interplay between the gut microbiome, immune regulation, and metabolic pathways offers novel perspectives in PDAC. Further research is warranted to validate these findings and translate them into clinical practice.

Nonetheless, several challenges persist within the current research landscape of PDAC. The pronounced molecular and phenotypic heterogeneity of PDAC contributes to the development of effective regimens. Moreover, PDAC patients frequently present with multiple comorbidities, which further exacerbates the complexity of treatment decision‐making and limits the applicability of standardized approaches. Consequently, future research must prioritize the integration of patient‐specific factors, including molecular profiles, comorbidities, and clinical characteristics, to develop tailored therapeutic strategies. Additionally, investigating optimized combination therapies that target multiple pathways simultaneously holds promise for enhancing treatment efficacy and improving patients’ quality of life.

In conclusion, research in PDAC is advancing rapidly, with the emergence of novel targeted therapies and immunotherapeutic approaches. However, overcoming critical barriers, including the challenges of personalized treatment, therapeutic resistance, and disease heterogeneity, remains imperative for the successful clinical translation of these strategies. To deliver optimal care for PDAC patients, an integrated, multidisciplinary approach that combines expertise from oncology, molecular biology, pharmacology, and supportive care will be essential. Such an approach will not only address the complexities of the disease but also ensure the efficacy of treatments.

## Author Contributions

Kexun Zhou and Hong Zhu contributed to the conception, design, and final approval of the submitted version. Literature was collected and analyzed by Kexun Zhou, Yingping Liu, and Chuanyun Tang. Kexun Zhou, Yingping Liu, and Chuanyun Tang contributed to the manuscript writing. Kexun Zhou contributed to the graphic design, and all authors conceived and approved the final manuscript.

## Ethics Statements

The authors have nothing to report.

## Conflicts of Interest

The authors declare that the study was conducted in the absence of any business or financial relationship that could be interpreted as a potential conflict of interest.

## Data Availability

Data sharing is not applicable to this article as no new data were created or analyzed in this study.
